# Perceived dignity is an unrecognized source of emotional distress in patients with rheumatic diseases: Results from the validation of the Mexican version of the Patient Dignity Inventory

**DOI:** 10.1371/journal.pone.0289315

**Published:** 2023-08-04

**Authors:** Virginia Pascual-Ramos, Irazú Contreras-Yáñez, Maximiliano Cuevas-Montoya, Guillermo A. Guaracha-Basáñez, Cesar Mario García-Alanís, Oscar Rodríguez-Mayoral, Harvey Max Chochinov

**Affiliations:** 1 Department of Immunology and Rheumatology, Instituto Nacional de Ciencias Médicas y Nutrición Salvador-Zubirán (INCMyN-SZ), Mexico City, Mexico; 2 Department of Psychiatry, Instituto Nacional de Ciencias Médicas y Nutrición Salvador-Zubirán (INCMyN-SZ, Mexico City, Mexico; 3 Palliative Care Service, Instituto Nacional de Cancerología, México City, México; 4 Department of Psychiatry, University of Manitoba, Cancer Care Manitoba, Winnipeg, Canada; Universal Scientific Education and Research Network, CAMEROON

## Abstract

**Introduction:**

Dignity has rarely been explored in patients with rheumatic diseases (RMDs), which contrasts with patients´ observations that dignity is a relevant area for research focus. The study’s primary objective was to adapt and validate the Mexican version of the Patient Dignity Inventory (PDI-Mx) in patients with RMDs, and to estimate the proportion of patients with distress related to perceived dignity (DPD) assessed with the PDI-Mx.

**Methods:**

This cross-sectional study was developed in 2 phases. Phase 1 consisted of pilot testing and questionnaire feasibility (n = 50 patients), PDI-Mx content validity (experts’ agreement), construct validity (exploratory factor analysis), discriminant validity (Heterotrait-Monotrait correlations’ rate [HTMT]), criterion validity (Spearman correlations) and PDI-Mx reliability with internal consistency (Cronbach’s alpha) and test-retest (intra-class correlation coefficients [ICC]) in 220 additional outpatients (among whom 30 underwent test-retest). Phase 2 consisted of quantifying DPD (PDI-Mx cut-off ≥54.4) in 290 outpatients with RMDs.

**Results:**

Overall, patients were representative of typical outpatients with RMDs from a National tertiary care level center. The 25-item PDI-Mx was found feasible, valid (experts’ agreement ≥82%; a 4-factor structure accounted for 68.7% of the total variance; HTMT = 0.608; the strength of the correlations was moderate to high between the PDI-Mx, the Depression, Anxiety, and Stress scale dimensions scores, and the Health Assessment Questionnaire Disability Index score) and reliable (Cronbach’s ɑ = 0.962, ICC = 0.939 [95%CI = 0.913–0.961]). DPD was present in 78 patients (26.9%).

**Conclusions:**

The PDI-Mx questionnaire showed good psychometric properties for assessing DPD in our population. Perceived dignity in patients with RMDs might be an unrecognized source of emotional distress.

## Introduction

The term dignity refers to a complex construct lacking definitional specificity, while theoretical analysis offers a range of dignity constructs [1–4]. The word is used in various ways and settings, and many classifications of multiple and even contradictory uses have been offered in the scientific literature [3, 4]. Dignity has been described as a fundamental feature of human beings, related to human rationality and morality, that does not depend on or vary with circumstances [4, 5]. This notion of dignity is recognized as intrinsic, human, basic, or absolute dignity [1]. However, within the healthcare context, dignity implicates how patients perceive themselves and perceive they are seen by others and how the nature of the illness affects the person’s life and identity [5–8]. It is generally equated with the person’s sense of autonomy and control and is affected by social interaction [5–8]. This notion of dignity is termed extrinsic, social, merit, or relative dignity [1] and has been logically and linguistically subordinate to intrinsic dignity [4].

Instruments assessing dignity from adult patients’ perspectives have been widely employed in palliative care, end-of-life care settings, acute care settings, and other groups of patients suffering from a wide variety of primarily chronic diseases [1, 8–20]. A recent review described the psychometric properties of eleven self-reported instruments used in different clinical settings [21]. It confirmed that the Patient Dignity Inventory (PDI), which was developed based on an empirical model of dignity in the terminally ill, has satisfactory psychometric properties. It is the most widely used instrument to assess distress related to (perceived) dignity (DPD) [1, 21]. The scale was first developed in terminally ill cancer patients [12, 13], although its use has extended to non-cancer patients. It has been validated across various populations in different countries [21]. There is a Mexican version (PDI-Mx) validated in 290 patients with cancer, integrated with 25 items; each one is scored on a five-point Likert scale. The 25 items are distributed into four factors (loss of meaning in life, anxiety and loss of autonomy, dependency, and social support), accounting for 64.7% of the total variance [22].

Perceived dignity might be affected by the medical care environment, along with patients feeling a diminished sense of worth or value and a burden to others [1, 10–14, 17–19]. Constitutional symptoms, such as fatigue and weakness, can further undermine autonomy and heighten physical dependency. These are well recognized in the clinical spectrum of patients with rheumatic diseases (RMDs). However, perceived dignity has rarely been explored [23], which contrasts with Swedish RMDs patients’ observations that dignity, identity, and quality of life are relevant areas to focus research [24]. In Rheumatology, international organizations have highlighted the necessity of incorporating patients’ perspectives into clinical decisions to embrace the patient-centered care model, recognized as the optimum healthcare model for chronic diseases [25].

With the above considerations in mind, the study’s objectives were to adapt and validate the PDI-Mx in patients with RMDs, and to estimate the proportion of patients with DPD assessed with the PDI-Mx.

## Materials and methods

The current study conforms to STROBE´s guidelines (Please refer to the S1 Appendix “STROBE checklist for cross-sectional studies”).

### Study design

This cross-sectional study was performed between January 2022 and July 2022. It was registered on trial.gov (NCT05248464). Guidelines for translation, adaptation, and validation of instruments or scales used in cross-cultural health care research [26] were followed.

Two phases were considered during study development.

Phase 1 consisted of adapting the PDI-Mx previously validated in Mexican patients with cancer [22] to Mexican patients with RMDs, followed by its validity and reliability. The PDI-Mx is a validated Mexican cancer patients’ version of the original PDI, developed in Canadian patients with cancer (Please refer to the S2 Appendix "Original Patient Dignity Inventory"). Each of the 25 items is scored on a five-point Likert scale, where 1 corresponds to "not a problem," and 5 is "an overwhelming problem. The PDI-Mx final score ranges from 25 to 125; higher scores indicate more DPD. Phase 1 included pilot testing for PDI-Mx content validity and feasibility, followed by PDI-Mx construct/criterion validity and reliability.

Phase 2 assessed DPD in the target population and applied the previously validated version of the PDI-Mx to consecutive outpatients with RMDs.

### Setting

The Instituto Nacional de Ciencias Médicas y Nutrición (INCMyN-SZ) belongs to the National Institutes of Health of Mexico. It is a tertiary care and national referral center for RMDs in Mexico City. In December 2020, total healthcare coverage (medications, medical assistance, laboratories, and diagnostic imaging studies) was officially adopted in the country, although its implementation has been challenging. Previously (and currently for some patients), patients had a spectrum of Federal government health coverage depending on their socioeconomic status, which is determined by social workers after a patient interview and an income-to-needs ratio assessment. Patients from the whole country can be referred, although most are from the metropolitan area.

The outpatient clinic of the Department of Immunology and Rheumatology database identified about 5000 patients with RMDs with at least one visit to the outpatient clinic during 2019. The ten most frequent diagnoses were Systemic Lupus Erythematosus (SLE) (33%), Rheumatoid Arthritis (RA) (31.6%), Systemic Sclerosis (4.8%), Systemic Vasculitis (4.4%), Primary Sjögren Syndrome (PSS), Spondyloarthritis (3.5%), Inflammatory Myopathies and Primary Antiphospholipid Syndrome (3% each), Mixed Connective Tissue Disease (1.9%), and Adult-onset Still Disease (0.6%).

Annually, the outpatient clinic is staffed by 11 board-certified rheumatologists and 8–10 rheumatology residents. A single primary rheumatologist is maintained during the entire patient’s follow-up, except for patients assigned to trainees in rheumatology, who change their primary physician every two years (training program duration).

### Participants

Participants were recruited at the Department of Immunology and Rheumatology outpatient clinic. Consecutive patients were identified while waiting for a scheduled consultation and approached by a co-investigator who briefly explained the study’s aim and explored patients’ interest in participating and ability to provide written informed consent. The informed consent process was performed once patients confirmed their interest (95% of the patients invited agreed to participate in the study). Inclusion criteria considered were patients with a definite rheumatologic diagnosis according to their primary rheumatologist criteria and who provided written informed consent. The exclusion criterion was patients in palliative care.

### Sample description and sample size estimation

Three convenience samples of different outpatients were used, and quotes (number of patients with a particular RMD diagnosis) were considered to represent the ten most frequent RMD diagnoses distribution in the outpatient clinic from the Department of Immunology and Rheumatology.

Two samples were considered during phase 1: The first one (S-1) included 50 patients who participated in pilot testing and PDI-Mx feasibility. The second sample (S-2) included 220 additional outpatients (8.8:1 respondent-to-item ratio) [26]; Both samples were used for PDI-Mx validation.

The third sample (S-3) included 290 patients with RMDs and was used to estimate the proportion of patients with DPD assessed with the PDI-Mx. We consider a prevalence of 13%, according to data from Solomon et al. [15] in patients with Chronic Obstructive Pulmonary Disease (COPD). Before the pandemic, 7.627 patients were coded with a definite rheumatic diagnosis within the Department of Immunology and Rheumatology’s database. The final estimated sample size was (at least) 262 patients, with a 95% confidence level and 4% of effect size or precision (Formula: $n=\left[\frac{N*{Z\alpha }^{2}*pq}{d^{2}*(N-1)+{Z\alpha }^{2}*pq}\right]$)

### Data collection and procedures

Three categories of data were collected during both phases.

The first category consisted of data related to relevant sociodemographic variables, RMDs-related variables, previous hospitalizations, treatment related-variables, and mental health-related variables (**Table 1**). Two data abstractors retrieved them from chart review and used standardized formats.

**Table 1:** Description of the patients.

	S-1	S-2	S-3
	n = 50	n = 220	n = 290
**Sociodemographic characteristics**			
Female sex*	41 (82)	179 (81.4)	237 (81.7)
Years of age	45.5 (34–59)	48 (35.3–60)	46 (35–55)
Years of formal education	12 (9–16)	12 (9–16)	12 (9–16)
Formal and non-formal job*	25 (50)	113 (51.4)	148 (51)
Married or Living with a partner*	21 (42)	119 (54.1)	152 (52.4)
Religious beliefs*	42 (84)	166 (75.5)	221 (76.2)
Medium-low socioeconomic status*	43 (86)	284 (83.6)	251 (86.6)
Family APGAR score (0–10 scale)	Not Done	9 (8–10)	9 (8–10)
Patients with normal family function*	Not Done	192 (87.3)	253 (87.2)
**Disease related-variables**			
Patients with SLE diagnosis*	13 (26)	68 (30.9)	91 (31.4)
Patients with RA diagnosis*	21 (42)	73 (33.2)	97 (33.4)
Patients with other diagnoses*	16 (32)	79 (35.9)	102 (35.2)
Years of disease duration	13 (7.8–20)	11 (6–20)	11 (6–19)
RAPID-3 score (0–30 scale)	Not Done	8.3 (2–15.2)	8 (2–15.3)
Patients with adequate disease activity control^1^*	Not Done	177 (80.5)	244 (84.1)
HAD-DI score (0–3 scale)	Not Done	0.13 (0–1.13)	0.13 (0–1)
Patients with disability (HAQ-DI score >0.5)*	Not done	102 (46.4)	132 (45.5)
Rheumatic Diseases Comorbidity Index score	0 (0–1)	0 (0–1)	0 (0–1)
Patients with ≥ 1 comorbid condition*	27 (54)	120 (54.5)	160 (55.2)
One year-previous hospitalizations*	9 (18)	50 (22.7)	72 (24.8)
Number of previous hospitalizations/patients^2^	1 (1–1)	1 (1–1)	1 (1–1)
**Treatment-related variables**			
Immunosuppressive treatment*	45 (90)	157 (71.4)	211 (72.8)
N° of immunosuppressive drugs /patient^2^	1 (1–2)	1 (1–2)	1 (1–2)
Glucocorticoids use*	18 (36)	91 (41.4)	117 (40.3)
**Mental health-related variables**			
Previous mental health-related comorbidity*	8 (16)	50 (22.7)	71 (24.5)
DASS21 score of ≥moderate severity*^,^^3^			
*Depression*	Not Done	50 (22.7)	69 (22.4)
*Anxiety*	Not Done	75 (34.1)	100 (34.5)
*Stress*	Not Done	52 (23.6)	67 (23.1)
Brief Resilient Coping Scale score (4–20 scale)	Not done	15 (12–18)	15 (12–18)
PDI-Mx score (25–125)	34.5 (28–48.3)	39.5 (29–57)	40 (29.8–58)

Data presented as median (Q25-Q75) as otherwise indicated.

*Number (%) of patients.

^1^According to the primary rheumatologist.

^2^Among those who met the characteristic

^3^Lovibond SH, Lovibond PF. Manual for the Depression Anxiety & Stress Scales. 2nd ed. Sydney: Psychology Foundation; 1995. S = Sample. APGAR = Adaptation Partnership Growth Affection Resolve. SLE = Systemic Lupus Erythematosus. RA = Rheumatoid Arthritis. RAPID-3 = Routine Assessment of Patient Index Data RAPID-3. HAQ-DI = Health Assessment Questionnaire Disability Index. DASS21 = Depression, Anxiety, and Stress Scale. PDI-Mx = Patient Dignity Inventory (Mexican version).

The second category consisted of data related to the following self-reported (unless patients required assistance) questionnaires and scales, which had a specific and standardized order: at first, the PDI-Mx (Please refer to the S3 Appendix "PDI-Mx"), then the Depression, Anxiety, and Stress scale (DASS21) [27], the Health Assessment Questionnaire Disability Index (HAQ-DI) [28], the family Adaptation Partnership Growth Affection Resolve (APGAR) [29], the Brief Resilient Coping Scale scores [30] and the Routine Assessment of Patient Index Data RAPID-3 [31].

The third category of data was retrieved from the attending primary rheumatologist, who defined the level of disease activity using a standardized format and scored the Rheumatic Diseases Comorbidity Index [32].

#### Phase 1 procedures

All phase 1 procedures were performed in S-2, but pilot testing and PDI-Mx feasibility were performed in S-1.

PDI-Mx feasibility was examined in the 50 patients participating in pilot testing according to the following criteria: the convenient time required to fill the questionnaire and the patient’s format acceptance, while the number of incomplete questionnaires was recorded.

Judgment experts determined PDI-Mx content validity [33]. Eleven experts (four rheumatologists, four bioethicists, two mental health specialists, and one social worker) independently rated the 25 items according to the presence or absence of relevance, appropriate wording, and meaning. Experts also rated the domains’ face validity and clarity of instructions. Experts were selected based on their experience with patients with RMDs and in questionnaires evaluation. A similar process was followed in 50 patients with RMDs during pilot testing to confirm content validity.

Finally, a brief questionnaire was applied to experts and patients to delineate the time period referenced in the instructions when answering the PDI-Mx (the original instructions referred to "in the last few days…").

Construct validity was evaluated using factorial analysis [33].

Discriminant validity was evaluated based on the ratio of the correlation between the PDI-Mx score and the RAPID-3 score [31, 34].

Criterion (concurrent) validity was examined according to correlations between the PDI-Mx score and the DASS21 [27], the HAQ-DI [28], the APGAR [29], and the Brief Resilient Coping Scale [30] scores [33]. In addition, all the patients with DASS21 scores of at least moderate severity for anxiety and depression had a psychiatric interview within one week by a certified mental health specialist to confirm clinically relevant symptoms of emotional distress and provide management.

Reliability was assessed with internal consistency and temporal stability (test-retest), which was tested after the questionnaire was applied to 30 patients, twice, at baseline and one week later [33].

#### Phase-2 procedures

The (previously validated version of the) PDI-Mx was administered along with additional questionnaires and scales previously described. Standardized formats were used to retrieve all the relevant information that is summarized in **Table 1**.

### Statistical analysis

The PDI-Mx score was calculated as the sum of the individual item’s score (min 25-Max 125). Receiving Operating Curve (ROC) was used to define the best PDI-Mx cut-off for DPD, with the gold standard defined according to DASS21 scores of at least moderate severity for anxiety and depression and a psychiatric interview confirming clinically relevant emotional distress symptoms. Patients with PDI-Mx scores above the cut-off were defined as with DPD.

Descriptive statistics were performed to describe PDI-Mx feasibility and the variables of the patients included in the three samples, with frequencies and percentages for categorical variables or the mean/median and standard deviation (SD)/Q25-Q75 for continuous variables with normal/non-normal distribution.

Appropriate tests were used to compare variables between groups (SLE vs. RA patients): The X^2^ test for categorical variables and the Mann-Whitney U test for continuous variables (non-normal distribution).

Content validity was examined with agreement percentages. Lawshe/Tristan’s content validity ratio was calculated for individual items [35] and the PDI-Mx (mean of individuals’ content validity ratios). Construct validity was evaluated using exploratory factorial analysis (principal components). Discriminant validity was analyzed using the Heterotrait-Monotrait ratio of correlation (HTMT) [34]. Criterion validity was analyzed using the Spearman rank correlation coefficient (rho) [36]. Cronbach’s α was used to assess the internal consistency of the questionnaire. For temporal stability/test-retest, intra-class correlation coefficients (ICC) and their 95% confidence intervals (CI) were calculated using a single measurement, absolute-agreement, 2-way mixed-effects model. Cronbach’s α, ICC, and 95% CI interpretations followed published recommendations [37]. Finally, the floor and ceiling effects of the questionnaire were determined as the percentage of patients who achieved the lowest and highest score on the scale, respectively.

Missing data were below 1%, and no imputation was performed.

All data collected in standardized formats were entered into an Excel database tailored to the current study. Data were exported to Statistical Package for the Social Sciences version 21.0 (SPSS Chicago IL) and further analyzed. A value of p<0.05 was considered statistically significant.

### Definitions

Content validity [33] is the extent that measurement instrument items are relevant and representative of the target population.

Construct validity [33] refers to how well a concept, idea, or behavior (a construct) is transformed into a functioning and operating reality.

Discriminant validity [33] is the extent that measures of different constructs diverge or are minimally correlated with one another.

Criterion concurrent validity [33] is the extent that a measure simultaneously relates to another measure that is supposed to relate.

Internal consistency [33] is the extent to which a measurement of a phenomenon provides stable and consistent results.

Test-retest reliability [26] is the ability of the scores of an instrument to be reproducible if it is used on the same patient while the patient´s condition has not changed.

### Ethical considerations

The Research Ethics Committee of the Instituto Nacional de Ciencias Médicas y Nutrición Salvador-Zubirán (INCMyN-SZ) approved the study (Reference number: IRE-4031-22-23-1). Patients provided written informed consent. The study was performed in compliance with the Helsinki Declaration [38].

Only one co-author responsible for database integrity accessed information that could identify individual participants. (Please refer to the S4 Appendix "PLOS’ questionnaire on inclusivity in global research")

## Results

### Patients characteristics

There were 50 patients in S-1, 220 in S-2, and 290 in S-3.

**Fig 1:**
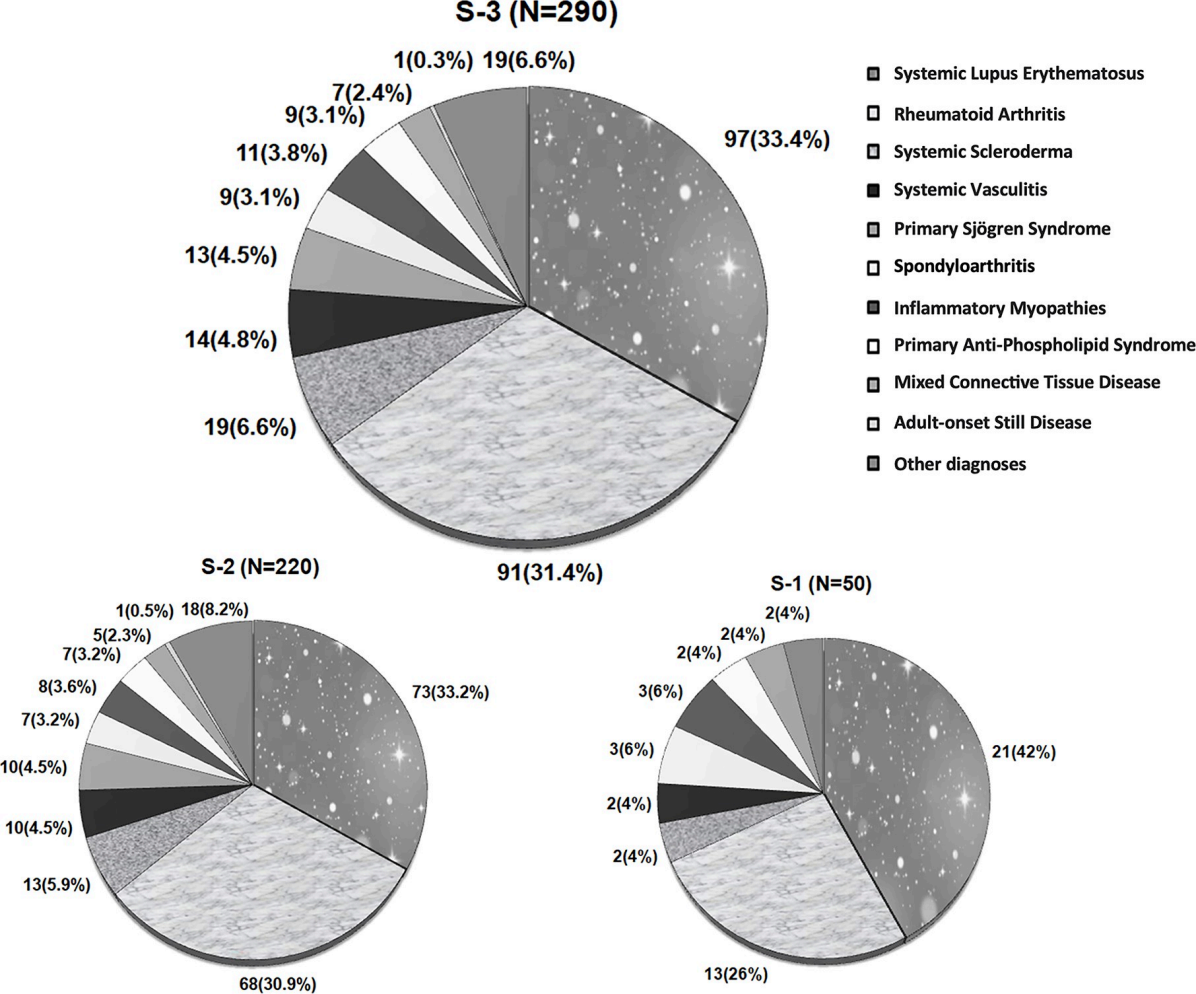
Diagnoses distribution in the three samples used.

**Fig 1** and **Table 1** summarize diagnoses distribution and patients’ characteristics in the samples for the PDI-Mx validation process (S-1 and S-2) and in the sample to estimate the proportion of patients with DPD (S-3). Overall, diagnoses quotes were represented, and the most frequent diagnoses were systemic lupus erythematosus (SLE) and rheumatoid arthritis (RA). Patients were primarily middle-aged women, working, living with a partner, acknowledged religious beliefs (85% Catholics), and had normal family function based on the family APGAR score. Overall, patients had substantial disease duration, adequate control of the disease activity of the underlying rheumatic disease, and HAQ-DI score that translated into the absence of disability. Regarding treatment, most patients received immunosuppressive drugs, and a significant proportion received additional glucocorticoids. Finally, a substantial proportion of the patients had previous mental health comorbidity and current psychological comorbidity/trauma of at least moderate severity [39]. Diagnoses distribution and patients’ characteristics were similar across samples (**Table 1**).

### PDI-Mx validation

#### PDI-Mx feasibility

All patients were willing to fill out the PDI-Mx, and 49 (98%) agreed on the format. Patients took (mean ± SD) 1.9±0.6 minutes to complete it.

#### PDI-Mx content validity

**Fig 2:**
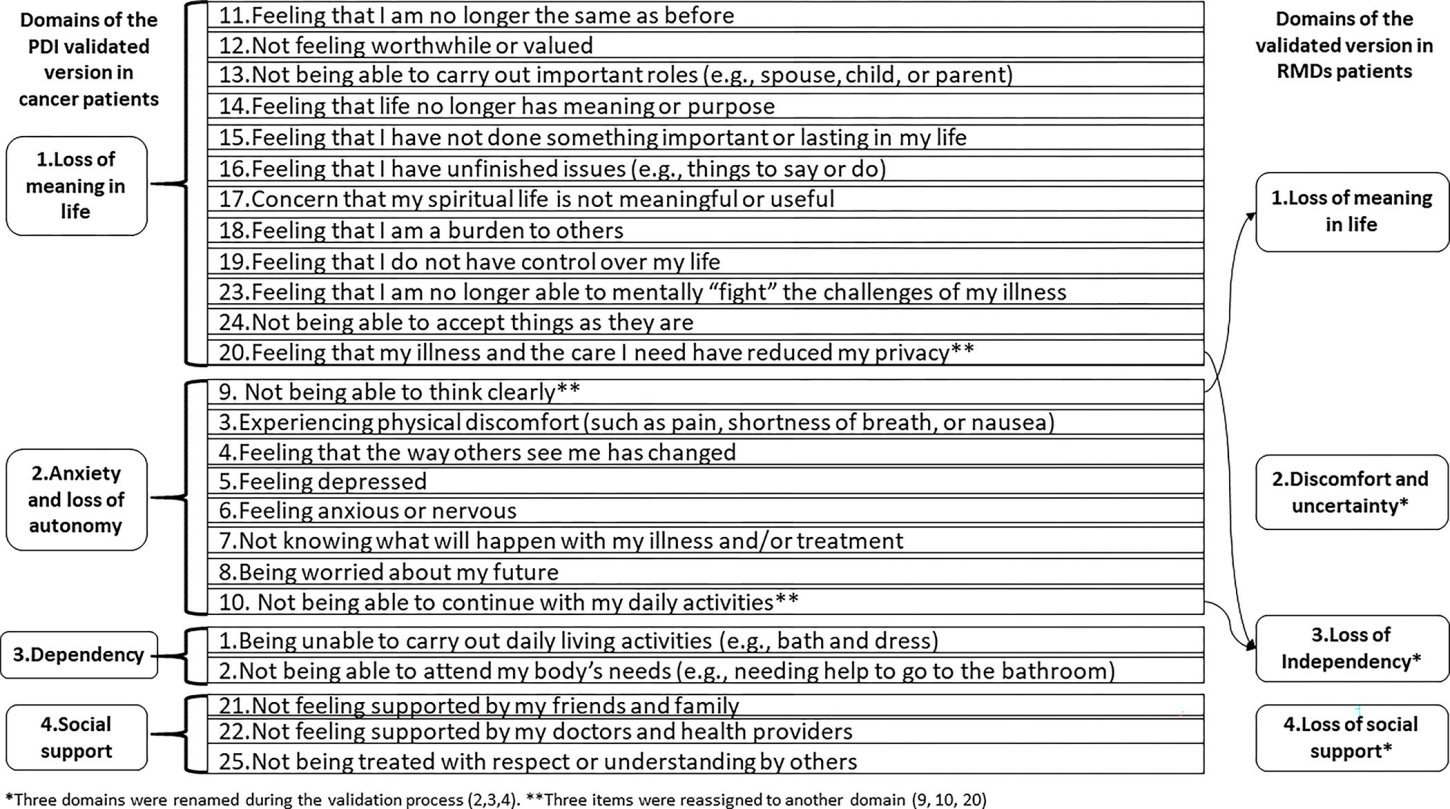
PDI-Mx structure pre and post-factorial analysis.

Experts agreed on the items and scale response evaluation (≥82% agreement), face validity (≥82% agreement), and instructions clarity (≥ 91% agreement). The (mean) content validity ratio for the PDI-Mx was 0.84. One expert suggested adding "and others" at the end of items 1, 3, and 13. (**Fig 2**).

During pilot testing, patients agreed on items and instructions clarity (≥ 86% and 94% agreement, respectively) and to add “and other” at the end of item-1 (n = 35 [70%]), item-3 (n = 32 [64%]) and item-13 (n = 28 [56%]).

Four experts agreed on "in the last four weeks" as the instructions period, which was also endorsed by 16 patients (32%). The mental health specialist involved in the study design also suggested this period based on published questionnaires related to emotional distress [40].

Patients’ and experts’ suggestions were all adopted.

#### PDI-Mx construct validity

The structure of the PDI-Mx underwent mild modifications after factorial analysis, as shown in **Fig 2**, where three items were charged in a different factor/domain. The 25 items were distributed into four domains named as follows: "Loss of meaning in Life" (Domain I), "Discomfort and uncertainty" (Domain II), "Loss of independence" (Domain III), and "Loss of social support" (Domain IV). The KMO measure of 0.945 and significant result (X^2^ = 4333.21, p≤0.001) for the Bartlett sphericity test confirmed the adequacy of the sample. A 4-factor structure was extracted, which accounted for 68.7% of the total variance. All factors had eigen values >1. The factors were equivalent to the four domains.

#### PDI-Mx discriminant validity

The HTMT ratio correlation between the PDI-Mx and RAPID-3 scores was 0.608.

#### PDI-Mx criterion validity

**Table 2** summarizes Spearman rank correlation coefficients (rho) between the PDI-Mx score (and the four domains scores), and specific dimensions scores of the DASS21 (depression, anxiety, and stress), the HAQ-DI score, the family APGAR score, and the Brief Resilient Coping Scale score. Overall, the strength of the correlations was moderate to high and consistently significant between the PDI-Mx score, the DASS21 dimensions scores, and the HAQ-DI score.

**Table 2:** Strength of the association between the PDI-Mx score, the DASS21 score, the HAQ-DI score, the family APGAR score, and the brief resilient coping scale score.

	DASS21	DASS21	DASS21		Family	
	*Anxiety*	*Depression*	*Stress*	HAQ-DI	APGAR	BRCS
**PDI-Mx global**	0.683	0.778	0.722	0.561	-0.309	-0.307
**Domain I**	0.690	0.779	0.695	0.539	-0.321	-0.307
**Domain II**	0.637	0.722	0.722	0.450	-0.260	-0.261
**Domain III**	0.509	0.525	0.485	0.640	NS	-0.176
**Domain IV**	0.525	0.584	0.520	0.465	-0.442	-0.219

DASS21 = Depression, Anxiety, and Stress Scale. HAQ-DI = Health Assessment Questionnaire Disability Index. APGAR = Adaptation Partnership Growth Affection Resolve. BRCS = Brief Resilient Coping Scale. PDI-Mx = Patient Dignity Inventory (Mexican version). NS = Not Significant. Domain I = Loss of meaning in Life. Domain II = Discomfort and uncertainty. Domain III = Loss of Independence. Domain IV = Loss of Social support.

#### PDI-Mx internal consistency and test-retest (Reliability)

Results of internal consistency (Cronbach’s α) and test-retest (ICC and 95% CI) of the PDI-Mx and each domain are presented in **Table 3**, which also presents floor and ceiling effects. The mean (±SD) of the time between the two measurements in the test-retest analysis was 6±0.8 days.

**Table 3:** PDI-Mx internal consistency, reliability/temporal stability, and floor and ceiling effects.

	Cronbach´s α	ICC (95% CI)*	Floor/ceiling effect (%)
PDI-Mx	0.962	0.939 (0.913–0.961)	6.4/0
Domain-1 *Loss of Meaning in Life*	0.949	0.903 (0.860–0.938)	19.1/0
Domain-2 *Discomfort and uncertainty*	0.882	0.814 (0.728–0.882)	8.6/0
Domain-3 *Loss of independence*	0.850	0.733 (0.604–0.831)	38.2/0.5
Domain-4 *Loss of social support*	0.729	0.4 (0.1–0.625)	59.1/0

ICC = Intraclass Correlation coefficient.CI = Confidence Interval.

*Limited to 30 patients.

### Prevalence of DPD in patients with RMDs

We first established the PDI-Mx cut-off to define DPD. In S-2, 79 patients had DASS21 depression and anxiety scores of at least moderate severity. All were referred for a psychiatric interview, although only 32 attended. The best PDI-Mx cut-off for DPD was 54.5.

Among the patients who integrated S-3, the median (Q25-Q75) PDI-MX score was 40 (29.8–58) (Minimum 25-Maximum 125). Seventy-eight patients scored PDI-Mx ≥54.5. Accordingly, 26.9% (95% CI: 21.8–32) of the patients had DPD, and the percentage was similar in the two most frequent RMD diagnoses: 27 out of 97 SLE patients (27.8% [95%CI: 18.8–36.9]) vs. 24 out of 91 RA patients (26.4% [95%CI:17.2–35.6]), p = 0.870.

**Table 4** summarizes PDI-Mx global and domain scores in the whole population and their comparison between SLE and RA patients; no differences were identified.

**Table 4:** PDI-Mx global and domains scores in the whole population and their comparison between SLE and RA patients.

	Global population, n = 290	SLE patients, n = 97	RA patients, n = 91	p
**PDI-Mx global score (Min 25-Max 125)**	40 (29.8–58)	41 (30–58)	37 (29–58)	0.347
**"Loss of meaning in life" (Min 12-Max 60)**	18 (13–26.3)	21 (14–26.5)	16 (13–25)	0.244
**"Discomfort and uncertainty” (Min 6-Max 30)**	12 (8–17)	12 (8–17)	11 (8–16)	0.840
**"Loss of independence" (Min 4-Max 20)**	5 (4–8)	5 (4–7)	5 (4–8)	0.294
**"Loss of social support" (Min 3-Max 15)**	3 (3–5)	3 (3–4)	3 (3–5)	0.705

SLE = Systemic Lupus Erythematosus. RA = Rheumatoid Arthritis PDI-Mx =.

## Discussion

The United Nations’ call for the right to health emphasizes a reciprocal relationship between health and dignity and that "health is a prerequisite to living a life in dignity" (General Comment No. 14, 2000) [41]. The scientific literature highlights a consistent and strong correlation between perceived dignity and quality of life, one of the most relevant outcomes from rheumatic patients’ perspectives [42]. Accordingly, the current paper, which focuses on validating the PDI-Mx in patients with RMDs and quantifying DPD, contributes to the knowledge of the impact of RMDs on patients’ biographies.

We first observed that the PDI-Mx had adequate psychometric properties to evaluate DPD in patients with RMDs. The validation process was rigorous and critical indicators of its quality were content, construct, discriminant, criterion concurrent validity, and reliability, evaluated with internal consistency and test-retest, as recommended [36]. Validity components were previously defined, and the mandatory validity tests were performed with suggested techniques [33]. The PDI-Mx showed adequate internal consistency. Cronbach´s α coefficient for the total scale and the majority of individual dimensions were good to excellent, and the test-retest reliability showed an ICC indicating good reliability. Moderate-to-high correlations between the PDI-Mx and additional questionnaires assessing emotional distress affirmed criterion validity [36]. Discriminant validity was convenient based on the HTMT ratio below 0.85 [34]. The PDI-Mx showed neither floor nor ceiling effect, defined when more than 15% of the patients achieved the lowest or highest score, respectively; floor and ceiling effects can reduce the possibility of detecting change over time [36]. The wording for items and instructions was optimized through face validity testing with input from a multidisciplinary group of healthcare providers and patients, who are the experts for reporting subjective outcomes [42]. Different samples of consecutive outpatients with RMDs, which were representative of real-world outpatients attending a tertiary care level center, were used for analysis, and accordingly, we consider our results could be generalized to other populations of patients with RMDs. After the validation, the PDI-Mx structure was primarily maintained and revealed a four-factor structure that explained 68.7% of the total variance. Only three items shifted to a different factor/domain compared to their distribution previous to the factorial analysis. Domains were named considering the importance of losses during rheumatic patients’ biographies. The changes in the structure might be explained by the evidence that culture influences a person’s sense of dignity, which highlights the need for cross-cultural research [6, 17]. In addition, an individual´s dignity is connected with his/her interactions with others in society, and relationships are culturally shaped and bound [6].

Second, the current study is the first to describe DPD among patients with RMDs, which contrasts with RMDs patients’ interests in dignity as an important area for research focus [24]. In rheumatology, we identified only two qualitative descriptions of perceived threatened dignity in women with fibromyalgia [23, 43]. The prevalence of DPD in our study was surprisingly high, particularly when compared to the 5–10% of the patients in palliative care in whom a “fractured” sense of dignity has been identified [9]. Additional studies about patients´ perceived loss of dignity in some non-cancer populations have also shown a lower prevalence than ours. Solomon et al. [15] detected a problematic loss of dignity in 13% of the patients with advanced stages of COPD. Chochinov et al. [14] compared the landscape of dignity-related distress across advanced COPD, Amyotrophic Lateral Sclerosis, End-Stage Renal Disease, and the frail institutionalized elderly using the PDI; loss of dignity was detected in 4% to 11% of the patients and did not differ significantly across these populations. However, a recent systematic review of patient-reported dignity and dignified care during acute hospital admission highlights a higher prevalence of moderate to severe loss of dignity in Asian patients [1]. In particular, two Chinese studies of patients with cancer reported up to 22% of moderate to severe loss of dignity assessed with the PDI [44, 45]. Oosterveld-Vlug et al. [46] did not report a prevalence of DPD; instead, they described the presence and influence of symptoms and experiences that undermine dignity in a group of nursing home residents, most of whom were affected by heart diseases, RA, stroke, and diabetes. Additional studies have also addressed threats to dignity in patients with dementia [20], heart failure [18], the critically ill [16], and acute inpatients [17], although the prevalence is not described. Our reported prevalence of DPD might reflect methodological differences across studies related to scales and definition of critical thresholds, unique cultural and socio-economic conditions associated with patients’ sense of dignity [2, 6, 7], and the additional collateral damage of the COVID-19 pandemic on emotional health [47]. Public life in Latin America has been characterized by fragile health systems and long-standing and pervasive inequity, which has led to a humanitarian crisis during the COVID-19 pandemic, particularly affecting vulnerable populations [20]. Also, various potential RMDs’ clinical expressions ultimately shape the biography of the patients; some are so pervasive that they contribute to multilayered stress and impact patients´ perceived dignity [48].

Finally, we did observe neither a different DPD prevalence nor a different pattern of DPD between SLE and RA patients. Although the study was not designed to reveal such differences, it might be argued that the full spectrum of RMDs’ clinical expression provides evidence that overall, a significant percentage of the patients might present with progressive health decline, disability, and distressing symptoms affecting their capacity to continue with usual activities and social roles, and limiting autonomy, all of which threaten patients’ perceived dignity [1, 2, 6, 7, 46, 49].

The study has various shortcomings that must be addressed. First, participants were recruited from a single academic, tertiary care health system in an urban center; additionally, we sampled different RMDs diagnoses, though two of them were highly represented (SLE and RA), which is representative of our outpatient clinic but may differ from other centers; this limits the generalizability of our findings. Second, outpatients had primarily long-standing disease and disease activity under control, suggesting that participants may only partially represent the full spectrum of RMDs phenotypes. Third, the order of questionnaires´ application was standardized, which may have impacted their compliance and attention. Fourth, RAPID-3 was used to assess disease activity/severity among patients with various rheumatic diagnoses, although the scale has been formally validated only in RA patients. Finally, the study took place during the COVID-19 pandemic, which might have affected the results.

## Conclusions

The current study informed the PDI-Mx rigorous validation process and described its critical quality indicators of validity and reliability, while the questionnaire’s feasibility was also confirmed. The study additionally revealed that DPD was homogeneously present in a significant percentage of Mexican patients with different RMDs, up to 27%. Notably, the complex theoretical construct of DPD was assessed with the previously validated questionnaire in the target population. Patients´ characteristics represented typical RMD outpatients, which thus favors the study results´ external validity.

We recommend that DPD be investigated in RMD patients. The PDI-Mx can inform whom patients should be referred for additional mental health evaluation and intervention to alleviate emotional discomfort. The study highlights that the impact of RMDs on patients’ lives surpasses the biomedical aspects of illness experiences. Further research may elucidate factors associated with DPD and the potential role of dignity-based interventions in treating and preventing severe psychological symptoms in patients with RMDs.

## Supporting information

S1 Appendix STROBE checklist for cross sectional studies. (PDF)

S2 Appendix Original Patient Dignity Inventory. (PDF)

S3 Appendix PDI-Mx. (PDF)

S4 Appendix PLOS’ questionnaire on inclusivity in global research. (PDF)
