# Supplementary material for: Perceived dignity is an unrecognized source of emotional distress in patients with rheumatic diseases: Results from the validation of the Mexican version of the Patient Dignity Inventory
Source: PLoS One. 2023 Aug 4;18(8):e0289315. doi: 10.1371/journal.pone.0289315 (PMC10403073; doi:10.1371/journal.pone.0289315)
Supplement: S2 Appendix — (PDF) [file pone.0289315.s002.pdf]

Patient Dignity Inventory

For each item, please indicate how much of a problem or concern these have been for you within the last few days

1. Not being able to carry out tasks associated with daily living (e.g., washing myself, getting dressed).
1☐ Not a problem 2☐ A slight problem 3☐ A problem 4☐ A major problem 5☐ An overwhelming problem

2. Not being able to attend to my bodily functions independently (e.g., needing assistance with toileting-related activities)
1☐ Not a problem 2☐ A slight problem 3☐ A problem 4☐ A major problem 5☐ An overwhelming problem

3. Experiencing physically distressing symptoms (such as pain, shortness of breath, nausea).
1☐ Not a problem 2☐ A slight problem 3☐ A problem 4☐ A major problem 5☐ An overwhelming problem

4. Feeling that how I look to others has changed significantly.
1☐ Not a problem 2☐ A slight problem 3☐ A problem 4☐ A major problem 5☐ An overwhelming problem

5. Feeling depressed.
1☐ Not a problem 2☐ A slight problem 3☐ A problem 4☐ A major problem 5☐ An overwhelming problem

6. Feeling anxious.
1☐ Not a problem 2☐ A slight problem 3☐ A problem 4☐ A major problem 5☐ An overwhelming problem

7. Feeling uncertain about my illness and treatment.
1☐ Not a problem 2☐ A slight problem 3☐ A problem 4☐ A major problem 5☐ An overwhelming problem

8. Worrying about my future.
1☐ Not a problem 2☐ A slight problem 3☐ A problem 4☐ A major problem 5☐ An overwhelming problem

9. Not being able to think clearly.
1☐ Not a problem 2☐ A slight problem 3☐ A problem 4☐ A major problem 5☐ An overwhelming problem

10. Not being able to continue with my usual routines.
1☐ Not a problem 2☐ A slight problem 3☐ A problem 4☐ A major problem 5☐ An overwhelming problem

11. Feeling like I am no longer who I was.
1☐ Not a problem 2☐ A slight problem 3☐ A problem 4☐ A major problem 5☐ An overwhelming problem

12. Not feeling worthwhile or valued.
1☐ Not a problem 2☐ A slight problem 3☐ A problem 4☐ A major problem 5☐ An overwhelming problem

13. Not being able to carry out important roles (e.g., spouse, parent).
1☐ Not a problem 2☐ A slight problem 3☐ A problem 4☐ A major problem 5☐ An overwhelming problem

14. Feeling that life no longer has meaning or purpose.
1☐ Not a problem 2☐ A slight problem 3☐ A problem 4☐ A major problem 5☐ An overwhelming problem

15. Feeling that I have not made a meaningful and lasting contribution during my lifetime.
1☐ Not a problem 2☐ A slight problem 3☐ A problem 4☐ A major problem 5☐ An overwhelming problem

16. Feeling I have 'unfinished business' (e.g., things left unsaid, or incomplete)
1☐ Not a problem 2☐ A slight problem 3☐ A problem 4☐ A major problem 5☐ An overwhelming problem

17. Concern that my spiritual life is not meaningful.
1☐ Not a problem 2☐ A slight problem 3☐ A problem 4☐ A major problem 5☐ An overwhelming problem

18. Feeling that I am a burden to others.
1☐ Not a problem 2☐ A slight problem 3☐ A problem 4☐ A major problem 5☐ An overwhelming problem

19. Feeling that I don't have control over my life.
1☐ Not a problem 2☐ A slight problem 3☐ A problem 4☐ A major problem 5☐ An overwhelming problem

20. Feeling that my illness and care needs have reduced my privacy.
1☐ Not a problem 2☐ A slight problem 3☐ A problem 4☐ A major problem 5☐ An overwhelming problem

21. Not feeling supported by my community of friends and family.
1☐ Not a problem 2☐ A slight problem 3☐ A problem 4☐ A major problem 5☐ An overwhelming problem

22. Not feeling supported by my health care providers.
1☐ Not a problem 2☐ A slight problem 3☐ A problem 4☐ A major problem 5☐ An overwhelming problem

23. Feeling like I am no longer able to mentally 'fight' the challenges of my illness.
1☐ Not a problem 2☐ A slight problem 3☐ A problem 4☐ A major problem 5☐ An overwhelming problem

24. Not being able to accept the way things are.
1☐ Not a problem 2☐ A slight problem 3☐ A problem 4☐ A major problem 5☐ An overwhelming problem

25. Not being treated with respect or understanding by others.
1☐ Not a problem 2☐ A slight problem 3☐ A problem 4☐ A major problem 5☐ An overwhelming problem
