# Supplementary material for: Perceived dignity is an unrecognized source of emotional distress in patients with rheumatic diseases: Results from the validation of the Mexican version of the Patient Dignity Inventory
Source: PLoS One. 2023 Aug 4;18(8):e0289315. doi: 10.1371/journal.pone.0289315 (PMC10403073; doi:10.1371/journal.pone.0289315)
Supplement: S3 Appendix — (PDF) [file pone.0289315.s003.pdf]

Patient Dignity Inventory in Mexican Cancer Patients (v. Mexico)
English version

For each statement, please indicate with a ✔ to what degree this has been a problem or concern in the past few days.

	Not a problem	A slight problem	A moderate problem	A serious problem	A very serious problem
1. Being unable to carry out daily living activities (e.g., bath, dress).	1	2	3	4	5
2. Not being able to attend my body's needs (e.g., needing help to go to the bathroom).	1	2	3	4	5
3. Experiencing physical discomfort (such as pain, shortness of breath, nausea).	1	2	3	4	5
4. Feeling that the way others see me has changed.	1	2	3	4	5
5. Feeling depressed.	1	2	3	4	5
6. Feeling anxious or nervous.	1	2	3	4	5
7. Not knowing what will happen with my illness and/or treatment.	1	2	3	4	5
8. Being worried about my future.	1	2	3	4	5
9. Not being able to think clearly.	1	2	3	4	5
10. Not being able to continue with my daily activities.	1	2	3	4	5
11. Feeling that I am no longer the same as before.	1	2	3	4	5
12. Not feeling worthwhile or valued.	1	2	3	4	5
13. Not being able to carry out important roles (e.g., spouse, child, parent or others).	1	2	3	4	5
14. Feeling that life no longer has meaning or purpose.	*1*	*2*	*3*	*4*	*5*

15. Feeling that I have not done something important or lasting in my life.	1	2	3	4	5
16. Feeling that I have unfinished issues (e.g., things to say or do).	1	2	3	4	5
17. Concern that my spiritual life is not meaningful or useful.	1	2	3	4	5
18. Feeling that I am a burden to others.	1	2	3	4	5
19. Feeling that I do not have control over my life.	1	2	3	4	5
20. Feeling that my illness and the care I need have reduced my privacy.	1	2	3	4	5
21. Not feeling supported by my friends and family.	1	2	3	4	5
22. Not feeling supported by my doctors and health providers.	1	2	3	4	5
23. Feeling that I am no longer able to mentally “fight” the challenges of my illness.	1	2	3	4	5
24. Not being able to accept things as they are.	1	2	3	4	5
25. Not being treated with respect or understanding by others.	1	2	3	4	5

Inventario de la Dignidad del Paciente
(Validado en pacientes con cáncer)
Spanish version for Mexico

Para cada enunciado, por favor, indique con una ✔ hasta qué grado esto ha sido un problema o preocupación en los últimos días.

	**No es un problema**	**Es un problema leve**	**Es un problema moderado**	**Es un problema grave**	**Es un problema muy grave**
1. No poder llevar a cabo actividades de la vida diaria (por ej., bañarme, vestirme).	1	2	3	4	5
2. No poder atender las necesidades de mi cuerpo (por ej., necesitar ayuda para ir al baño).	1	2	3	4	5
3. Sentir malestar físico (como dolor, falta de aire, náusea).	1	2	3	4	5
4. Sentir que ha cambiado la forma en la que me ven los demás.	1	2	3	4	5
5. Sentirme deprimido(a).	1	2	3	4	5
6. Sentirme ansioso(a) o nervioso(a).	1	2	3	4	5
7. No saber qué va a pasar con mi enfermedad y/o tratamiento.	1	2	3	4	5
8. Estar preocupado(a) por mi futuro.	1	2	3	4	5
9. No poder pensar con claridad.	1	2	3	4	5
10. No poder continuar con mis actividades diarias.	1	2	3	4	5
11. Sentir que ya no soy él/la mismo(a) que antes	1	2	3	4	5
12. No sentirme valioso(a) o valorado(a).	1	2	3	4	5
13. No ser capaz de llevar a cabo roles importantes (por ej. esposo(a), hijo(a), madre/padre).	1	2	3	4	5
14. Sentir que la vida ya no tiene significado o propósito.	*1*	*2*	*3*	*4*	*5*

15. Sentir que no he hecho algo importante o duradero en mi vida.	1	2	3	4	5
16. Sentir que tengo “pendientes” (por ej., cosas por decir o hacer).	1	2	3	4	5
17. Preocupación de que mi vida espiritual no es significativa o útil.	1	2	3	4	5
18. Sentir que soy una carga para los demás.	1	2	3	4	5
19. Sentir que no tengo control sobre mi vida.	1	2	3	4	5
20. Sentir que mi enfermedad y los cuidados que necesito han reducido mi privacidad.	1	2	3	4	5
21. No sentirme apoyado(a) por mis amigos y familiares.	1	2	3	4	5
22. No sentirme apoyado(a) por mis médicos y personal de la salud.	1	2	3	4	5
23. Sentir que ya no puedo “luchar” mentalmente con los retos de mi enfermedad.	1	2	3	4	5
24. No poder aceptar las cosas como son.	1	2	3	4	5
25. No ser tratado(a) con respeto o comprensión por los demás.	1	2	3	4	5
