# Supplementary material for: Perceived dignity is an unrecognized source of emotional distress in patients with rheumatic diseases: Results from the validation of the Mexican version of the Patient Dignity Inventory
Source: PLoS One. 2023 Aug 4;18(8):e0289315. doi: 10.1371/journal.pone.0289315 (PMC10403073; doi:10.1371/journal.pone.0289315)
Supplement: Attachment — Submitted filename: Responses to the academic editor.docx [file pone.0289315.s006.docx]

**Responses to the academic editor (DPD-PONE-D-23-05892-R1).**

1. Lines 150 to 151: The sentence should be rephrased.

***Response.*** ***We have rephrased the sentence.***

2. Line 232: "later" instead of "latter".

***Response.*** ***We have followed the editor´s indication.***

3. Line 333: "eigen values".

***Response. We have followed the editor´s indication.***

4. Lines 453-457: rephrase the sentence "First, participants...our findings."

***Response. We have rephrased the paragraph.***

5. Line 477: biomedical or biological?

***Response. We consider either term acceptable.***

6. Please, provide the 95% confidence intervals of your proportions of DPD

***Response. We have added 95% CI.***

7. Between the conclusion and references sections, there are no statements about conflicts of interests, authors contributions, funding and acknowledgments in the manuscript.

***Response. We have added the statements required.***
