# Supplementary material for: Perceived dignity is an unrecognized source of emotional distress in patients with rheumatic diseases: Results from the validation of the Mexican version of the Patient Dignity Inventory
Source: PLoS One. 2023 Aug 4;18(8):e0289315. doi: 10.1371/journal.pone.0289315 (PMC10403073; doi:10.1371/journal.pone.0289315)
Supplement: Peer review history

# Peer review: Decision Letter 0

**DOI:** 10.1371/journal.pone.0289315.r001

**Authors:** Mickael Essouma

5 May 2023

PONE-D-23-05892Perceived dignity in patients with rheumatic diseases: an unrecognized source of emotional distress.PLOS ONE

Dear Dr. Pascual-Ramos,

Thank you for submitting your manuscript to PLOS ONE. After careful consideration, we feel that it has merit but does not fully meet PLOS ONE’s publication criteria as it currently stands. Therefore, we invite you to submit a revised version of the manuscript that addresses the points raised during the review process. Please submit your revised manuscript by Jun 19 2023 11:59PM. If you will need more time than this to complete your revisions, please reply to this message or contact the journal office at plosone@plos.org. When you're ready to submit your revision, log on to https://www.editorialmanager.com/pone/ and select the 'Submissions Needing Revision' folder to locate your manuscript file.

Please include the following items when submitting your revised manuscript:

- A rebuttal letter that responds to each point raised by the academic editor and reviewer(s). You should upload this letter as a separate file labeled 'Response to Reviewers'.
- A marked-up copy of your manuscript that highlights changes made to the original version. You should upload this as a separate file labeled 'Revised Manuscript with Track Changes'.
- An unmarked version of your revised paper without tracked changes. You should upload this as a separate file labeled 'Manuscript'.

If you would like to make changes to your financial disclosure, please include your updated statement in your cover letter. Guidelines for resubmitting your figure files are available below the reviewer comments at the end of this letter.

If applicable, we recommend that you deposit your laboratory protocols in protocols.io to enhance the reproducibility of your results. Protocols.io assigns your protocol its own identifier (DOI) so that it can be cited independently in the future. For instructions see: https://journals.plos.org/plosone/s/submission-guidelines#loc-laboratory-protocols. Additionally, PLOS ONE offers an option for publishing peer-reviewed Lab Protocol articles, which describe protocols hosted on protocols.io. Read more information on sharing protocols at https://plos.org/protocols?utm_medium=editorial-email&utm_source=authorletters&utm_campaign=protocols.

We look forward to receiving your revised manuscript.

Kind regards,

Mickael Essouma, M. D.

Academic Editor

PLOS ONE

Journal Requirements:

When submitting your revision, we need you to address these additional requirements.

1. Please ensure that your manuscript meets PLOS ONE's style requirements, including those for file naming. The PLOS ONE style templates can be found at 

https://journals.plos.org/plosone/s/file?id=wjVg/PLOSOne_formatting_sample_main_body.pdf and 

https://journals.plos.org/plosone/s/file?id=ba62/PLOSOne_formatting_sample_title_authors_affiliations.pdf

2. Please include a complete copy of PLOS’ questionnaire on inclusivity in global research in your revised manuscript. Our policy for research in this area aims to improve transparency in the reporting of research performed outside of researchers’ own country or community. The policy applies to researchers who have travelled to a different country to conduct research, research with Indigenous populations or their lands, and research on cultural artefacts. The questionnaire can also be requested at the journal’s discretion for any other submissions, even if these conditions are not met.  Please find more information on the policy and a link to download a blank copy of the questionnaire here: https://journals.plos.org/plosone/s/best-practices-in-research-reporting. Please upload a completed version of your questionnaire as Supporting Information when you resubmit your manuscript.

3. We noticed you have some minor occurrence of overlapping text with the following previous publication(s), which needs to be addressed:

- https://doi.org/10.1371/journal.pone.0236167

- https://doi.org/10.1016/j.socscimed.2006.08.039

In your revision ensure you cite all your sources (including your own works), and quote or rephrase any duplicated text outside the methods section. Further consideration is dependent on these concerns being addressed.

4. We note that you have stated that you will provide repository information for your data at acceptance. Should your manuscript be accepted for publication, we will hold it until you provide the relevant accession numbers or DOIs necessary to access your data. If you wish to make changes to your Data Availability statement, please describe these changes in your cover letter and we will update your Data Availability statement to reflect the information you provide.

Additional Editor Comments:

The authors are commendable for a landmark (first ever) study of the dignity of patients with rheumatic diseases using a validated and reliable (Lam et al J Adv Nurs. 2022;78(12):3952-3973. doi: 10.1111/jan.15436) dignity questionnaire (PDI) developed and previously translated in Mexican (Rodriguez-Maroyal et al J Palliat Med. 2021;24(11):1626-1633. doi: 10.1089/jpm.2020.0572) authors as well. However, the great work is not clearly reported. It therefore needs a major revision before it can be accepted for publication. It should be clear in the manuscript that you neither developed (except if you have added some questions specific to RMD patients, that you will therefore specify), neither translated the PDI-Mx as this had been done previously, but you were only validating it in an RMD population as it has not been done before and there are expected variation from a disease group to another (Tsang et al. Saudi J Anaesth

. 2017 May;11(Suppl 1):S80-S89. doi: 10.4103/sja.SJA_203_17 and Sousa et al. J Eval Clin Pract. 2011 Apr;17(2):268-74. doi: 10.1111/j.1365-2753.2010.01434.x).

Major comments

The title should be revised. I proposed this one to reflect the two aspects of your work: "Dignity (qualify the dignity studied, as asked by the reviewer: human dignity? Social dignity? Lin et al. Nurs Ethics. 2013;20(2):168-77. doi: 10.1177/0969733012458609) of patients with Rheumatic diseases: results from the Mexican PDI".

The keywords should be revised as well in keeping with the title.

-Introduction: the first paragraph is irrelevant. Dignity is not so well known by medical health professionals whilst nurses are well used to that concept, so there is a need for many clarifications from the start of the manuscript. I suggest a one-page introduction split into three paragraphs: paragraph 1 on the definition of dignity, highlighting the lack of a consensual definition and mentioning the keywords that should be in the definition of dignity and the types of dignity: see for example the following reviews: Lin et al. Nurs Ethics. 2013;20(2):168-77. doi: 10.1177/0969733012458609; Xiao et al. Psychooncology

. 2021 Aug;30(8):1220-1231. doi: 10.1002/pon.5687; Torabizadeh et al. Hosp Top. 2021 Oct-Dec;99(4):187-197. doi: 10.1080/00185868.2021.1897487. In the second paragraph, you would discuss measurement methods of dignity in medicine to date (Lam et al J Adv Nurs. 2022;78(12):3952-3973. doi: 10.1111/jan.15436), explaining why the PDI is important and stating that there is already a validated mexican version but in cacer patients, you would also tell in this paragraph the contexts of common dignity studies in Medicine (diseases and persons who have mostly studied it: nurses. In the third paragraph, you would say that dignity is not studied in Rheumatology despite the fact that patients rate it among their priority topics (Bergsten et al. Musculoskeletal Care. 2014;12(3):194-7. doi: 10.1002/msc.1073), and patients perspectives has become an important concern in Rheumatology, then clearly state the aims of the study: validate the PDI-Mx in patients with RMDs, and estimate the proportion of patients with distress related to perceived dignity as assessed by the PDI-Mx.

Methods. You have uploaded the STROBE guidelines as supplementary material, but the first sentence of the Methods section should state that your report conforms to STROBE guidelines. Also, you did not follow the strobe guidelines that I invite you to follow. Notably, I recommend the following sub-sections for the methods section: Study design ( I agree with the cross-sectional, and add the study registration number from Clinical Trials.gov, as well as a statement that you complied with guidelines on developing, translating and validating questionnaires: Tsang et al. Saudi J Anaesth. 2017 May;11(Suppl 1):S80-S89. doi: 10.4103/sja.SJA_203_17 and Sousa et al. J Eval Clin Pract. 2011 Apr;17(2):268-74. doi: 10.1111/j.1365-2753.2010.01434.x), Setting (it is very important to well describe the setting in a cross-sectional study as this is used to assess the study quality: where is located; urban/rural? How many patients you see yearly at the outpatient service? How many rheumatologists? How many patients are registered in your database? Where do they come from: the whole country or from a single province? And so on), Participants (clear statement of inclusion [including the specification of the classification criteria used for each RMD]and exclusion criteria), Sample size estimation (I would say convenient sample size and delete the details on sample size you have mentioned as this is the first study in patients with RMDs, and there is no consensus on the appropriate sample size for a questionnaire validation: Tsang et al. Saudi J Anaesth

. 2017 May;11(Suppl 1):S80-S89. doi: 10.4103/sja.SJA_203_17), Data collection (it should be clear that you collected two types of data: data on RMDs and data on dignity with the PDI-Mx. So, you had one questionnaire besides the PDI-Mx, I guess. Specify the data on RMDs that you collected as well as the sources of information. Clearly describe the PDI-Mx items which should be uploaded as a supplementary material, and the procedure for collecting the data with the PDI-Mx and for validation procedures that involve study participants: pilot testing with initial validation and test-retest reliability), Statistical analyses (how you exported data from the questionnaires to the software, which software did you use for statistical analysis? Which statistical tests did you use to compute estimates on RMDs and proportion of distress related to perceived dignity, as well as for the validation processes, notably: internal consistency, test-retest reliability, inter-rater reliability, content validity, construct validity? How do you report qualitative and quantitative results, internal consistency (Cronbach alpha)...? How did you define statistical significance of results? Just to add that using the AUC in a questionnaire validation study is irrelevant, because this is not a diagnostic test accuracy study), Definitions (please, clarify here the definitions on construct, content validity, internal consistency, test-retest reliability used, of course based on guidelines: https://hal.science/hal-02546799/document), and then Ethical considerations .

Results. It should be split into two major parts (after the subsection of the general characteristics of the study population), as highlighted by a reviewer: Results of the PDI-Mx validation (internal consistency i.e., value of Cronchbach alpha, test-retest reliability [Pearsons correlation coefficient], inter-rater reliability [kappa], content validity, construct validity), and then results on distress related perceived dignity: not just the proportion of patients with distress-related perceived dignity, but also the factors associated with an abnormal PDI-Mx to further inform us (see for example Fuseini et al. J Adv Nurs

. 2022 Nov;78(11):3540-3558. doi: 10.1111/jan.15370).

Discussion. Summarize the main findings on both PDI-Mx validity and distress-related perceived dignity in patients with RMDs, discuss your results based on your experience and the literature on PDI-Mx, make feasible recommendations to Rheumatologists and other interested health professionals, and discuss the limitations and strengths of your study.

Conclusion. A clear, succint take-home message and perspectives should be provided.

Revise the abstract according to the full-text.

Free the manuscript from citation gaming (Macdonald. Social Science Information Volume 61, Issue 4, December 2022, Pages 457-480) as I will verify this in the revised manuscript, and update the references so that they include articles published within the last ten years, except for relevant articles from yourselves as you have made important contributions since many decades.

Minor comments

Please, delete the figure on RMDs proportions, and rather complete those data in the table on general characteristics of the study population that are commonly mentioned for RMDs as you know.

Some edits are necessary.

Make mostly short sentences and paragraphs (every new idea warrants a new paragraph), with long sentences and paragraphs representing only about 20% of the manuscript.

[Note: HTML markup is below. Please do not edit.]

Reviewers' comments:

Reviewer's Responses to Questions

**Comments to the Author**

1. Is the manuscript technically sound, and do the data support the conclusions?

The manuscript must describe a technically sound piece of scientific research with data that supports the conclusions. Experiments must have been conducted rigorously, with appropriate controls, replication, and sample sizes. The conclusions must be drawn appropriately based on the data presented. 

Reviewer #1: Yes

Reviewer #2: Yes

Reviewer #3: Yes

Reviewer #4: Yes

**********

2. Has the statistical analysis been performed appropriately and rigorously? 

Reviewer #1: Yes

Reviewer #2: I Don't Know

Reviewer #3: Yes

Reviewer #4: I Don't Know

**********

3. Have the authors made all data underlying the findings in their manuscript fully available?

The PLOS Data policy requires authors to make all data underlying the findings described in their manuscript fully available without restriction, with rare exception (please refer to the Data Availability Statement in the manuscript PDF file). The data should be provided as part of the manuscript or its supporting information, or deposited to a public repository. For example, in addition to summary statistics, the data points behind means, medians and variance measures should be available. If there are restrictions on publicly sharing data—e.g. participant privacy or use of data from a third party—those must be specified.

Reviewer #1: Yes

Reviewer #2: Yes

Reviewer #3: Yes

Reviewer #4: Yes

**********

4. Is the manuscript presented in an intelligible fashion and written in standard English?

PLOS ONE does not copyedit accepted manuscripts, so the language in submitted articles must be clear, correct, and unambiguous. Any typographical or grammatical errors should be corrected at revision, so please note any specific errors here.

Reviewer #1: Yes

Reviewer #2: Yes

Reviewer #3: Yes

Reviewer #4: No

**********

5. Review Comments to the Author

Please use the space provided to explain your answers to the questions above. You may also include additional comments for the author, including concerns about dual publication, research ethics, or publication ethics. (Please upload your review as an attachment if it exceeds 20,000 characters)

Reviewer #1: It seems to me a good work that explores a subject little explored in rheumatic diseases. I have some observations:

1) Is the dignity that you are measuring moral dignity (the patient's behavior in their environment) or real dignity, which is how patients are valued by other people?

2) Were there patients who participated in several phases or were there different patients in each phase?

3) You have a conceptual error in the methodology. Criterion validity and construct validity are basically the same. The difference is that in the criterion validity there is an alternative method of measuring the phenomenon under study; Therefore, comparisons can be made with other similar instruments (for example, in lupus there are several instruments to measure disease activity, so if a new one is available, criterion validity can be made by comparing it with others such as SLEDAI, BILAG or RIFLE that already have a proven validity). When there is no alternative method of measurement, then construct validity must be used and comparisons with phenomena that may be affected by the instrument being evaluated should be made. In this case, it seems, only construct validity should be carried out. Factorial analysis and principal components are performed to establish whether the instrument is one-dimensional or multidimensional and which items correspond to each domain (Clinimetrics-Feinstein).

4) as it is a multidimensional phenomenon (in the Spanish validation 3 dimensions were reported: psychological and existential distress, physical symptoms and dependency and social support and you report 4 dimensions: loss of meaning in life, discomfort and uncertainly, loss of independence and loss of social support) I believe that it is not necessary to establish a single cut-off point and have the measurements per domain (similar to the SF-36)

5) It would be interesting to know if the instrument has discriminative capacity

6) With what scale did you measure the socioeconomic level? When you refer to religious beliefs, what are you referring to?

7) It is more advisable to use the term glucocorticoids instead of corticosteroids (Buttgereit F, da Silva JA, Boers M, Burmester GR, Cutolo M, Jacobs J, Kirwan J, Köhler L, Van Riel P, Vischer T, Bijlsma JW. Standardised nomenclature for glucocorticoid dosages and glucocorticoid treatment regimens: current questions and tentative answers in rheumatology. Ann Rheum Dis. 2002 Aug;61(8):718-22. doi: 10.1136/ard.61.8.718).

Reviewer #2: Very interesting paper

Title of the paper, writing of the abstract and the paper itself dwell mainly on the item perceived dignity and situation in rheumatic diseases.

However, the validation of a scale in Mexican Spanish, complying with all requirements, is in itself a major issue and should on its own probably be the major point, then followed by the results in the RMD population. Could you elaborate why you did not do this? This would entail “turning around” your paper, validation of PDI Mex first and results in RMD population following.

Introduction: no description is made of the PDI which is going to be validated and used. Please describe.

Methods, sample size: why did you use the COPD data (13%) to estimate sample size instead of the validated data for cancer? Was the COPD data taken from a Mex validated scale or the US data as per Solomon et al. Does this influence possible results?

The whole paper describes extensively the validation of the PDI Mex in RMD.

The results after this, in RMD patients come later. They are interesting, limitations are well described (ie SLE and RA were the more frequent diseases although they are not clearly representative of a primary or secondary care clinic. Having said that, they are those which most commonly may affect perceived dignity).

In my view the validation process has been thorough and I think this should be the principal point and results should come after that. I would re write the paper with that in mind.

Something like: Validation of the Mexican version of the Patient Dignity Inventory (PDI-Mx in patients with rheumatic diseases: Perceived dignity, an unrecognized source of emotional distress.

Reviewer #3: The topic is very interesting and clinically relevant.

I think several points should be clarified for a better understanding:

1-I think that the questions of the original questionnaire should be detailed, which domain is taken into account and added as supplementary material

2-I think the sample size calculation formula should be added

3-Who administered the questionnaire?

4-Patients with previous psychiatric pathology were excluded? or psychiatric involvement secondary to a rheumatic disease, eg lupus?

5-In figure 1, the detail of each abbreviation should be

6-Figure 2, I think you need more detail in your explanation, the content is not understood, the differences in colors and the line mean something?

Reviewer #4: 1. In my review, I have only considered the conceptual aspects and the understandability of the manuscript. I do not have enough expertise to assess the methodological and statistical aspects of this study.

2. The interest and originality of this paper lies in the circumstance that it applies for the first time Chochinov’s Patient Dignity Inventory (DPI) developed to patients with rheumatic diseases to identify the frequency of distress related to perceived dignity among them. It is probably also the first time that the DPI is applied in Mexico.

3. In general, the paper is well and clearly written, and makes a valuable contribution to the understanding of patients’ sense of dignity.

I have only a few remarks:

1. The conclusion is too short and vague, and does not reflect adequately the richness of the study. I would suggest expanding the conclusion a little with some more detailed information about the study results (for example, that DPD is present in up to 27% of patients with rheumatic diseases; or the main factors of DPD).

2. Acronyms must be used consistently along the paper: the authors use at times “DPD” and at times “DRPD”.

3. I would suggest removing the adjective “current” when referring to the COVID-19 pandemic (lines 344 and 346), unless it is still prevalent in Mexico.

**********

6. PLOS authors have the option to publish the peer review history of their article (what does this mean?). If published, this will include your full peer review and any attached files.

If you choose “no”, your identity will remain anonymous but your review may still be made public.

**Do you want your identity to be public for this peer review?** For information about this choice, including consent withdrawal, please see our Privacy Policy.

Reviewer #1: No

Reviewer #2: **Yes: **Luis J. Catoggio, MD, PhD

Reviewer #3: No

Reviewer #4: No

**********

[NOTE: If reviewer comments were submitted as an attachment file, they will be attached to this email and accessible via the submission site. Please log into your account, locate the manuscript record, and check for the action link "View Attachments". If this link does not appear, there are no attachment files.]

While revising your submission, please upload your figure files to the Preflight Analysis and Conversion Engine (PACE) digital diagnostic tool, https://pacev2.apexcovantage.com/. PACE helps ensure that figures meet PLOS requirements. To use PACE, you must first register as a user. Registration is free. Then, login and navigate to the UPLOAD tab, where you will find detailed instructions on how to use the tool. If you encounter any issues or have any questions when using PACE, please email PLOS at figures@plos.org. Please note that Supporting Information files do not need this step.

# Author comment: Author response to Decision Letter 0

**DOI:** 10.1371/journal.pone.0289315.r002

15 Jun 2023

Responses to editor and reviewers (DPD-PONE-D-23-05892_R1)

Journal Requirements:

When submitting your revision, we need you to address these additional requirements.

1. Please ensure that your manuscript meets PLOS ONE's style requirements, including those for file naming. The PLOS ONE style templates can be found at 

https://journals.plos.org/plosone/s/file?id=wjVg/PLOSOne_formatting_sample_main_body.pdf and 

https://journals.plos.org/plosone/s/file?id=ba62/PLOSOne_formatting_sample_title_authors_affiliations.pdf

Response. We have followed the recommendation. 

2. Please include a complete copy of PLOS’ questionnaire on inclusivity in global research in your revised manuscript. Our policy for research in this area aims to improve transparency in the reporting of research performed outside of researchers’ own country or community. The policy applies to researchers who have travelled to a different country to conduct research, research with Indigenous populations or their lands, and research on cultural artefacts. The questionnaire can also be requested at the journal’s discretion for any other submissions, even if these conditions are not met. Please find more information on the policy and a link to download a blank copy of the questionnaire here: https://journals.plos.org/plosone/s/best-practices-in-research-reporting. Please upload a completed version of your questionnaire as Supporting Information when you resubmit your manuscript.

Response. We have uploaded a completed version of the questionnaire on inclusivity in global research.

3. We noticed you have some minor occurrence of overlapping text with the following previous publication(s), which needs to be addressed:

- https://doi.org/10.1371/journal.pone.0236167 (Development and validation of a questionnaire assessing household work limitations (HOWL-Q) in women with rheumatoid arthritis)

Response: We have reviewed the document (an article from our group), and the overlapping corresponds to ethical considerations (local IRB acceptance, provision of written informed consent, and study compliance with the Helsinki Declaration). We have rephrased it. 

- https://doi.org/10.1016/j.socscimed.2006.08.039 (Jacobson Nora. Dignity and health: A review)

In your revision ensure you cite all your sources (including your own works), and quote or rephrase any duplicated text outside the methods section. Further consideration is dependent on these concerns being addressed.

Response. The overlapping text was cited in the previous version. However, we have rephrased duplicated text. All the relevant sources have been cited, including all the references identified related to dignity in different populations, not just articles published in the last ten years. We highlight this point as the editor also request the following: “Free the manuscript from citation gaming (Macdonald. Social Science Information Volume 61, Issue 4, December 2022, Pages 457-480) as I will verify this in the revised manuscript, and update the references so that they include articles published within the last ten years, except for relevant articles from yourselves as you have made important contributions since many decades”. We hope our selection of references will satisfied the reviewer. 

4. We note that you have stated that you will provide repository information for your data at acceptance. Should your manuscript be accepted for publication, we will hold it until you provide the relevant accession numbers or DOIs necessary to access your data. If you wish to make changes to your Data Availability statement, please describe these changes in your cover letter and we will update your Data Availability statement to reflect the information you provide.

Response. We appreciate the editor's comment. We have made a change in the availability of data; the changes are explained in the cover letter.

Additional Editor Comments:

The authors are commendable for a landmark (first ever) study of the dignity of patients with rheumatic diseases using a validated and reliable (Lam et al J Adv Nurs. 2022;78(12):3952-3973. doi: 10.1111/jan.15436) dignity questionnaire (PDI) developed and previously translated in Mexican (Rodriguez-Maroyal et al J Palliat Med. 2021;24(11):1626-1633. doi: 10.1089/jpm.2020.0572) authors as well. However, the great work is not clearly reported. It therefore needs a major revision before it can be accepted for publication. It should be clear in the manuscript that you neither developed (except if you have added some questions specific to RMD patients, that you will therefore specify), neither translated the PDI-Mx as this had been done previously, but you were only validating it in an RMD population as it has not been done before and there are expected variation from a disease group to another (Tsang et al. Saudi J Anaesth . 2017 May;11(Suppl 1):S80-S89. doi: 10.4103/sja.SJA_203_17 and Sousa et al. J Eval Clin Pract. 2011 Apr;17(2):268-74. doi: 10.1111/j.1365-2753.2010.01434.x).

Response. We appreciate the editor's comments. In the study design section, we have been very explicit that we are adapting the questionnaire (PDI-Mx from cancer Mexican patients) to Mexican patients with RMDs and validating it. 

Major comments

The title should be revised. I proposed this one to reflect the two aspects of your work: "Dignity (qualify the dignity studied, as asked by the reviewer: human dignity? Social dignity? Lin et al. Nurs Ethics. 2013;20(2):168-77. doi: 10.1177/0969733012458609) of patients with Rheumatic diseases: results from the Mexican PDI".

Response. Based on the editor's and reviewers' suggestions, we have updated the title to "Perceived dignity is an unrecognized source of emotional distress in patients with rheumatic diseases: Results from the validation of the Mexican version of the Patient Dignity Inventory."

Regarding dignity qualification, we opted for "perceived dignity," which evokes the patient's perception of the dignity notion. The term is descriptive and in line with similar notions of dignity such as social dignity (Nora Jacobson 2007), attributed dignity (Sulmasy 2013), personal dignity (Leung, D., 2007. Granting death with dignity: patient, family and professional perspectives. International Journal of Palliative Nursing 13 (4), 170e174) and dynamic dignity (Rodríguez-Prat A et al (2016) Patient Perspectives of Dignity, Autonomy, and Control at the End of Life: Systematic Review and MetaEthnography. PLoS ONE 11(3): e0151435. doi:10.1371/journal.pone.0151435). It also permits the clear differentiation from notions of dignity, which can be identified in the literature as Human, intrinsic, and basic dignity. 

The keywords should be revised as well in keeping with the title.

Response. We proposed the following keywords: Perceived dignity, emotional distress, Patient Dignity Inventory validation, and rheumatic diseases. 

-Introduction: the first paragraph is irrelevant. Dignity is not so well known by medical health professionals whilst nurses are well used to that concept, so there is a need for many clarifications from the start of the manuscript. I suggest a one-page introduction split into three paragraphs: paragraph 1 on the definition of dignity, highlighting the lack of a consensual definition and mentioning the keywords that should be in the definition of dignity and the types of dignity: see for example the following reviews: Lin et al. Nurs Ethics. 2013;20(2):168-77. doi: 10.1177/0969733012458609; Xiao et al. Psychooncology . 2021 Aug;30(8):1220-1231. doi: 10.1002/pon.5687; Torabizadeh et al. Hosp Top. 2021 Oct-Dec;99(4):187-197. doi: 10.1080/00185868.2021.1897487. In the second paragraph, you would discuss measurement methods of dignity in medicine to date (Lam et al J Adv Nurs. 2022;78(12):3952-3973. doi: 10.1111/jan.15436), explaining why the PDI is important and stating that there is already a validated mexican version but in cacer patients, you would also tell in this paragraph the contexts of common dignity studies in Medicine (diseases and persons who have mostly studied it: nurses. In the third paragraph, you would say that dignity is not studied in Rheumatology despite the fact that patients rate it among their priority topics (Bergsten et al. Musculoskeletal Care. 2014;12(3):194-7. doi: 10.1002/msc.1073), and patients perspectives has become an important concern in Rheumatology, then clearly state the aims of the study: validate the PDI-Mx in patients with RMDs, and estimate the proportion of patients with distress related to perceived dignity as assessed by the PDI-Mx.

Response: We have tried our best to adopt the majority of the editor’s suggestions while also considering the proposal from reviewer 2, who request a description of the PDI in the introduction. 

Methods. You have uploaded the STROBE guidelines as supplementary material, but the first sentence of the Methods section should state that your report conforms to STROBE guidelines. 

Response. We have adopted the recommendation. 

Also, you did not follow the strobe guidelines that I invite you to follow. Notably, I recommend the following sub-sections for the methods section: Study design (I agree with the cross-sectional, and add the study registration number from Clinical Trials.gov, as well as a statement that you complied with guidelines on developing, translating and validating questionnaires: Tsang et al. Saudi J Anaesth. 2017 May;11(Suppl 1):S80-S89. doi: 10.4103/sja.SJA_203_17 and Sousa et al. J Eval Clin Pract. 2011 Apr;17(2):268-74. doi: 10.1111/j.1365-2753.2010.01434.x).

Response. The recommendation has been followed. 

Setting (it is very important to well describe the setting in a cross-sectional study as this is used to assess the study quality: where is located; urban/rural? How many patients you see yearly at the outpatient service? How many rheumatologists? How many patients are registered in your database? Where do they come from: the whole country or from a single province? And so on),

Response. The suggestion has been incorporated in the updated version. 

Participants (clear statement of inclusion [including the specification of the classification criteria used for each RMD]and exclusion criteria), … 

Response. The suggestion has been incorporated. A definite rheumatologic diagnosis was based on the primary rheumatologist criteria. No classification criteria were used. 

Sample size estimation (I would say convenient sample size and delete the details on sample size you have mentioned as this is the first study in patients with RMDs, and there is no consensus on the appropriate sample size for a questionnaire validation: Tsang et al. Saudi J Anaesth . 2017 May;11(Suppl 1):S80-S89. doi: 10.4103/sja.SJA_203_17), 

Response. We have revised and incorporated the reference suggested. We agree with the editor that no absolute rules exist for the sample size needed to validate a questionnaire. However, according to the reference, the respondent-to-item ratio can further strengthen the rationale for the sample size. Also, it is stated that a sample size of 200 is considered fair. Our updated proposal for the first sample size calculation (PDI-Mx validation) is a rational exercise we kindly request to maintain in the manuscript.

We additionally performed a second exercise of sample size calculation to estimate the proportion of patients with DPD assessed with the PDI-Mx. We used the data from Solomon et al. in patients with COPD, which was questioned by reviewer 2. We share with the editor our response. “Sample size calculation was challenging because this is the first study that evaluates DPD in patients with RMDs, and no data are available. Regarding the impact of a particular disease on patient-reported outcomes, we argue that COPD might behave similarly to RMDs, as both belong to chronic non-transmissible diseases. Meanwhile, a cancer diagnosis might have a different effect on patient-reported outcomes, probably driven by the mortality risk perceived by the patient. However, we also know that dignity is a cultural concept and that individual attitudes, values, and perceptions are related to maintaining dignity. The 13% estimation based on Solomon data might affect statistical inference power in our population of RMD patients. However, we finally obtained a higher prevalence, giving us a strength of over 80%.

Data collection (it should be clear that you collected two types of data: data on RMDs and data on dignity with the PDI-Mx. So, you had one questionnaire besides the PDI-Mx, I guess. Specify the data on RMDs that you collected as well as the sources of information. Clearly describe the PDI-Mx items which should be uploaded as a supplementary material, and the procedure for collecting the data with the PDI-Mx and for validation procedures that involve study participants: pilot testing with initial validation and test-retest reliability), 

Response. We have updated the whole section to comply with the editor´s requirements. 

Statistical analyses (how you exported data from the questionnaires to the software, which software did you use for statistical analysis? Which statistical tests did you use to compute estimates on RMDs and proportion of distress related to perceived dignity, as well as for the validation processes, notably: internal consistency, test-retest reliability, inter-rater reliability, content validity, construct validity? How do you report qualitative and quantitative results, internal consistency (Cronbach alpha)...? How did you define statistical significance of results? Just to add that using the AUC in a questionnaire validation study is irrelevant, because this is not a diagnostic test accuracy study), 

Response. We have complied with the editor's requirements. We agree with the editor’s comment about AUC utility in a questionnaire validation and have omitted the data. 

Definitions (please, clarify here the definitions on construct, content validity, internal consistency, test-retest reliability used, of course based on guidelines: https://hal.science/hal-02546799/document), and then Ethical considerations.

Response A” definitions” section has been added to the updated version of the manuscript. Recommended references have been reviewed and included. 

Results. It should be split into two major parts (after the subsection of the general characteristics of the study population), as highlighted by a reviewer: Results of the PDI-Mx validation (internal consistency i.e., value of Cronchbach alpha, test-retest reliability [Pearsons correlation coefficient], inter-rater reliability [kappa], content validity, construct validity), and then results on distress related perceived dignity: not just the proportion of patients with distress-related perceived dignity, but also the factors associated with an abnormal PDI-Mx to further inform us (see for example Fuseini et al. J Adv Nurs . 2022 Nov;78(11):3540-3558. doi: 10.1111/jan.15370).

Response. We have adopted the editor’s suggestions. However, we have not described factors associated with DPD as we might be unpowered to identify some potential associations. We have increased the sample size to over 400 patients with RMDs. We are analyzing their data to integrate a second paper (if/when the current article is approved) focusing on factors associated with DPD. Based on the following, we argue that the present article might be unpowered to detect related factors. Chochinov et al. (Lancet 2002) reported an effect size of 0.63 in quality of life scores among patients with cancer, in whom mean (SD) values were 4.4 (2.89) and 6.1 (2.5), respectively, in patients with a fractured sense of dignity and their counterparts. We arbitrarily hypothesized an effect size of 0.30 and estimated the sample size using a one-tailed test, 5% significance level, and 80% of power; we obtained 278 patients, and the number was increased to (at least) 334 patients considering 20% of losses for non-analyzable data. In addition, we believe the current version of the manuscript that describes the PDI-Mx validation process and the estimation of DPD in patients with RMDs has sufficient merit to be published. 

Discussion. Summarize the main findings on both PDI-Mx validity and distress-related perceived dignity in patients with RMDs, discuss your results based on your experience and the literature on PDI-Mx, make feasible recommendations to Rheumatologists and other interested health professionals, and discuss the limitations and strengths of your study.

Response. We propose an updated version of the “Discussion” section, and we have considered the editor´s recommendations. 

Conclusion. A clear, succinct take-home message and perspectives should be provided.

Response. We have updated the conclusions section considering the reviewer´s 4 suggestions as he/she made a few remarks. He/she suggested expanding the conclusion. We have also included practical recommendations for rheumatologists. 

Revise the abstract according to the full text.

Response. The abstract has been updated according to the changes included in the full text. 

Free the manuscript from citation gaming (Macdonald. Social Science Information Volume 61, Issue 4, December 2022, Pages 457-480) as I will verify this in the revised manuscript, and update the references so that they include articles published within the last ten years, except for relevant articles from yourselves as you have made important contributions since many decades.

Response. We appreciate the editor’s information regarding citation gaming, a concept we were unaware of. We have reviewed the reference and still don´t recognize citation gaming in the previous or current versions. We have, however, deleted a few references. 

Minor comments

Please, delete the figure on RMDs proportions, and rather complete those data in the table on general characteristics of the study population that are commonly mentioned for RMDs as you know.

Response. We want to keep the figure because it provides an easy visualization of the patient’s diagnoses quotes in the three samples. However, we have added to Table 1 three more lines (the table is already quite long) that include the number of patients with SLE, RA, and other diagnoses. Hopefully, this proposal will satisfy the editor. 

Some edits are necessary.

Response. We have revised the whole manuscript with an editing service. 

Make mostly short sentences and paragraphs (every new idea warrants a new paragraph), with long sentences and paragraphs representing only about 20% of the manuscript.

Response. We have made our best effort to comply with the editor´s requirements. 

 [Note: HTML markup is below. Please do not edit.]

Please use the space provided to explain your answers to the questions above. You may also include additional comments for the author, including concerns about dual publication, research ethics, or publication ethics. (Please upload your review as an attachment if it exceeds 20,000 characters)

Reviewer #1: 

It seems to me a good work that explores a subject little explored in rheumatic diseases. I have some observations:

Response. We appreciate the reviewer´s comment. 

1) Is the dignity that you are measuring moral dignity (the patient's behavior in their environment) or real dignity, which is how patients are valued by other people?

Response. We are measuring (distress related to) "perceived dignity," which evokes the patient's perception of the dignity notion. The term is descriptive and in line with similar notions of dignity such as social dignity (Nora Jacobson 2007), attributed dignity (Sulmasy 2013), personal dignity (Leung, D., 2007. Granting death with dignity: patient, family and professional perspectives. International Journal of Palliative Nursing 13 (4), 170e174) and dynamic dignity (Rodríguez-Prat A et al (2016) Patient Perspectives of Dignity, Autonomy, and Control at the End of Life: Systematic Review and MetaEthnography. PLoS ONE 11(3): e0151435. doi:10.1371/journal.pone.0151435). It also permits the clear differentiation from notions of dignity, which can be identified in the literature as Human, intrinsic, and basic dignity.

2) Were there patients who participated in several phases or were there different patients in each phase?

Response. Once we confirmed PDI-Mx validation without significant changes in the questionnaire, data from patients who participated in phase one were used in phase two. Patients from pilot testing only participate in phase one. 

3) You have a conceptual error in the methodology. Criterion validity and construct validity are basically the same. The difference is that in the criterion validity there is an alternative method of measuring the phenomenon under study; Therefore, comparisons can be made with other similar instruments (for example, in lupus there are several instruments to measure disease activity, so if a new one is available, criterion validity can be made by comparing it with others such as SLEDAI, BILAG or RIFLE that already have a proven validity). When there is no alternative method of measurement, then construct validity must be used and comparisons with phenomena that may be affected by the instrument being evaluated should be made. In this case, it seems, only construct validity should be carried out. Factorial analysis and principal components are performed to establish whether the instrument is one-dimensional or multidimensional and which items correspond to each domain (Clinimetrics-Feinstein).

Response. We appreciate the reviewer´s comment. However, we can´t conciliate her/his proposal with the academic editor’s requirement to include a specific reference and add a definition section. The reference suggested (updated as number 37) differentiates between criterion and construct validity, describes definitions for validity components, and describes the techniques suggested to provide shreds of evidence. We have adopted in the updated version all the recommendations. 

4) as it is a multidimensional phenomenon (in the Spanish validation 3 dimensions were reported: psychological and existential distress, physical symptoms and dependency and social support and you report 4 dimensions: loss of meaning in life, discomfort and uncertainly, loss of independence and loss of social support) I believe that it is not necessary to establish a single cut-off point and have the measurements per domain (similar to the SF-36). 

Response. We agree with the reviewer that DPD is a multidimensional phenomenon. The Mexican version validated in cancer patients, and the current PDI-Mx (validated in patients with RMDs) had four dimensions. We consider a single cut-off point establishes the presence (or absence) of DPD and has practical implications for rheumatologists. DPD (present vs. absent) accounts for (significant) distress related to perceived dignity, and conceptualizing the phenomenon as a dichotomous variable informs which patients should be referred for a psychiatric evaluation/intervention. Otherwise, clinicians might need clarification about translating a score into a clinical decision.

5) It would be interesting to know if the instrument has discriminative capacity.

Response. We have updated the manuscript (“Patient and methods” and “Results” sections) to describe discriminant validity. 

6) With what scale did you measure the socioeconomic level? When you refer to religious beliefs, what are you referring to?

Response. In the setting section, we added that ” ...patients had a spectrum of Federal government health coverage depending on their socioeconomic level, which social workers defined after patients’ interviews and income-to-needs ratio assessments.”

Religious beliefs were self-referred by the patients upon questioning. 

7) It is more advisable to use the term glucocorticoids instead of corticosteroids (Buttgereit F, da Silva JA, Boers M, Burmester GR, Cutolo M, Jacobs J, Kirwan J, Köhler L, Van Riel P, Vischer T, Bijlsma JW. Standardised nomenclature for glucocorticoid dosages and glucocorticoid treatment regimens: current questions and tentative answers in rheumatology. Ann Rheum Dis. 2002 Aug;61(8):718-22. doi: 10.1136/ard.61.8.718).

Response. The suggestion has been adopted. Thank you for the reference. 

Reviewer #2

Very interesting paper

Title of the paper, writing of the abstract and the paper itself dwell mainly on the item perceived dignity and situation in rheumatic diseases.

However, the validation of a scale in Mexican Spanish, complying with all requirements, is in itself a major issue and should on its own probably be the major point, then followed by the results in the RMD population. Could you elaborate why you did not do this? This would entail “turning around” your paper, validation of PDI Mex first and results in RMD population following. 

Response: We appreciate Dr. Luis J. Catoggio’s comments. The first version of the current manuscript was in line with your proposal. However, it was seriously criticized, and the editor from another journal finally rejected the manuscript because of the emphasis on the validation process, which was deemed unnecessary and irrelevant for rheumatologists. The results from the validation process were required to be located as supplemental material by one of the reviewers. I recognized that your revised version is the “reaction” to that academic experience. We have updated the whole manuscript to highlight the validation process. 

Introduction: no description is made of the PDI, which will be validated and used. Please describe.

Response. The suggestion has been adopted. 

Methods, sample size: why did you use the COPD data (13%) to estimate sample size instead of the validated data for cancer? Was the COPD data taken from a Mex validated scale or the US data as per Solomon et al. Does this influence possible results?

Response. Sample size calculation was challenging because this is the first study that evaluates DPD in patients with RMDs, and no data are available. Regarding the impact of a particular disease on patient-reported outcomes, we argue that COPD might behave similarly to RMDs, as both belong to chronic non-transmissible diseases. Meanwhile, a cancer diagnosis might have a different effect on patient-reported outcomes, probably driven by the mortality risk perceived by the patient. However, we also know that dignity is a cultural concept and that individual attitudes, values, and perceptions are related to maintaining dignity. The 13% estimation based on Solomon data might affect statistical inference power in our population of RMD patients. We finally obtained a higher prevalence, giving us a strength of over 80%.

The whole paper describes extensively the validation of the PDI Mex in RMD.

The results after this, in RMD patients come later. They are interesting, limitations are well described (ie SLE and RA were the more frequent diseases although they are not clearly representative of a primary or secondary care clinic. Having said that, they are those which most commonly may affect perceived dignity).

In my view the validation process has been thorough and I think this should be the principal point and results should come after that. I would re write the paper with that in mind.

Something like: Validation of the Mexican version of the Patient Dignity Inventory (PDI-Mx in patients with rheumatic diseases: Perceived dignity, an unrecognized source of emotional distress.

Response. We propose an updated title that includes and highlights the validation process as the central aspect of the manuscript to support the results: “Perceived dignity is an unrecognized source of emotional distress in patients with rheumatic diseases: Results from the validation of the Mexican version of the Patient Dignity Inventory.” The title proposed intends to integrate the suggestion of Dr. Luis J Catoggio and that of the academic editor. In addition, the whole manuscript has been updated to highlight the validation process while emphasizing the prevalence of this phenomenon in patients with rheumatic diseases. 

Reviewer #3: The topic is very interesting and clinically relevant.

Response: We appreciate the reviewer’s comment. 

I think several points should be clarified for a better understanding:

1-I think that the questions of the original questionnaire should be detailed, which domain is taken into account and added as supplementary material

Response: A brief description of the original instrument has been incorporated into the introduction section and added as supplementary material. In addition, Figure 2 has been updated to visualize items and their distribution into four domains in the original PDI-Mx (validated in cancer patients) and the PDI-Mx validated in patients with RMDs. 

2-I think the sample size calculation formula should be added.

Response. We have added the formula in the updated section. 

3-Who administered the questionnaire?

Response. We have clarified that the questionnaires applied, including the PDI-Mx, were self-reported unless patients required assistance. 

4-Patients with previous psychiatric pathology were excluded? or psychiatric involvement secondary to a rheumatic disease, eg lupus?

Response. They were not, as long as cognitive agency to provide informed consent was confirmed. We have added to the updated version. 

5-In Figure 1, the detail of each abbreviation should be added.

Response. We have adopted the suggestion. 

6-Figure 2, I think you need more detail in your explanation, the content is not understood, the differences in colors and the line mean something?

Response. We have updated Figure 2 and provided a brief explanation. 

Reviewer #4: 1. In my review, I have only considered the conceptual aspects and the understandability of the manuscript. I do not have enough expertise to assess the methodological and statistical aspects of this study.

2. The interest and originality of this paper lies in the circumstance that it applies for the first time Chochinov’s Patient Dignity Inventory (DPI) developed to patients with rheumatic diseases to identify the frequency of distress related to perceived dignity among them. It is probably also the first time that the DPI is applied in Mexico.

3. In general, the paper is well and clearly written and makes a valuable contribution to the understanding of patients’ sense of dignity.

Response. We highly appreciate the reviewer´s comments. 

I have only a few remarks:

1. The conclusion is too short and vague, and does not reflect adequately the richness of the study. I would suggest expanding the conclusion a little with some more detailed information about the study results (for example, that DPD is present in up to 27% of patients with rheumatic diseases; or the main factors of DPD).

Response. We propose an updated version of the conclusions. 

2. Acronyms must be used consistently along the paper: the authors use at times “DPD” and at times “DRPD”.

Response: We apologize for the mistake. The manuscript has been updated accordingly.

3. I would suggest removing the adjective “current” when referring to the COVID-19 pandemic (lines 344 and 346), unless it is still prevalent in Mexico.

Response. We have adopted the suggestion.

Attachment Submitted filename: Respons to reviewers.docx Click here for additional data file.

# Editor report: Decision Letter 1

**DOI:** 10.1371/journal.pone.0289315.r003

**Authors:** Mickael Essouma

12 Jul 2023

PONE-D-23-05892R1Perceived dignity is an unrecognized source of emotional distress in patients with rheumatic diseases: Results from the validation of the Mexican version of the Patient Dignity Inventory.PLOS ONE

Dear Dr. Pascual-Ramos,

Thank you for submitting your manuscript to PLOS ONE. After careful consideration, we feel that it has merit but does not fully meet PLOS ONE’s publication criteria as it currently stands. Therefore, we invite you to submit a revised version of the manuscript that addresses the points raised during the review process.

Please submit your revised manuscript by Aug 26 2023 11:59PM. If you will need more time than this to complete your revisions, please reply to this message or contact the journal office at plosone@plos.org. When you're ready to submit your revision, log on to https://www.editorialmanager.com/pone/ and select the 'Submissions Needing Revision' folder to locate your manuscript file.

Please include the following items when submitting your revised manuscript:

- A rebuttal letter that responds to each point raised by the academic editor and reviewer(s). You should upload this letter as a separate file labeled 'Response to Reviewers'.
- A marked-up copy of your manuscript that highlights changes made to the original version. You should upload this as a separate file labeled 'Revised Manuscript with Track Changes'.
- An unmarked version of your revised paper without tracked changes. You should upload this as a separate file labeled 'Manuscript'.

If you would like to make changes to your financial disclosure, please include your updated statement in your cover letter. Guidelines for resubmitting your figure files are available below the reviewer comments at the end of this letter.

If applicable, we recommend that you deposit your laboratory protocols in protocols.io to enhance the reproducibility of your results. Protocols.io assigns your protocol its own identifier (DOI) so that it can be cited independently in the future. For instructions see: https://journals.plos.org/plosone/s/submission-guidelines#loc-laboratory-protocols. Additionally, PLOS ONE offers an option for publishing peer-reviewed Lab Protocol articles, which describe protocols hosted on protocols.io. Read more information on sharing protocols at https://plos.org/protocols?utm_medium=editorial-email&utm_source=authorletters&utm_campaign=protocols.

We look forward to receiving your revised manuscript.

Kind regards,

Mickael Essouma, M. D.

Academic Editor

PLOS ONE

Journal Requirements:

Please review your reference list to ensure that it is complete and correct. If you have cited papers that have been retracted, please include the rationale for doing so in the manuscript text, or remove these references and replace them with relevant current references. Any changes to the reference list should be mentioned in the rebuttal letter that accompanies your revised manuscript. If you need to cite a retracted article, indicate the article’s retracted status in the References list and also include a citation and full reference for the retraction notice.

Additional Editor Comments:

Lines 150 to 151: The sentence should be rephrased.

Line 232: "later" instead of "latter".

Line 333: "eigen values".

Lines 453-457: rephrase the sentence "First, participants...our findings."

Line 477: biomedical or biological?

Please, provide the 95% confidence intervals of your proportions of DPD

Between the conclusion and references sections, there are no statements about conflicts of interests, authors contributions, funding and acknowledgments in the manuscript.

[Note: HTML markup is below. Please do not edit.]

[NOTE: If reviewer comments were submitted as an attachment file, they will be attached to this email and accessible via the submission site. Please log into your account, locate the manuscript record, and check for the action link "View Attachments". If this link does not appear, there are no attachment files.]

While revising your submission, please upload your figure files to the Preflight Analysis and Conversion Engine (PACE) digital diagnostic tool, https://pacev2.apexcovantage.com/. PACE helps ensure that figures meet PLOS requirements. To use PACE, you must first register as a user. Registration is free. Then, login and navigate to the UPLOAD tab, where you will find detailed instructions on how to use the tool. If you encounter any issues or have any questions when using PACE, please email PLOS at figures@plos.org. Please note that Supporting Information files do not need this step.

# Author comment: Author response to Decision Letter 1

**DOI:** 10.1371/journal.pone.0289315.r004

14 Jul 2023

Responses to the academic editor (DPD-PONE-D-23-05892-R1).

1. Lines 150 to 151: The sentence should be rephrased.

Response. We have rephrased the sentence. 

2. Line 232: "later" instead of "latter".

Response. We have followed the editor´s indication.

3. Line 333: "eigen values".

Response. We have followed the editor´s indication.

4. Lines 453-457: rephrase the sentence "First, participants...our findings."

Response. We have rephrased the paragraph. 

5. Line 477: biomedical or biological?

Response. We consider either term acceptable. 

6. Please, provide the 95% confidence intervals of your proportions of DPD

Response. We have added 95% CI. 

7. Between the conclusion and references sections, there are no statements about conflicts of interests, authors contributions, funding and acknowledgments in the manuscript.

Response. We have added the statements required.

Attachment Submitted filename: Responses to the academic editor.docx Click here for additional data file.

# Editor report: Decision Letter 2

**DOI:** 10.1371/journal.pone.0289315.r005

**Authors:** Mickael Essouma

17 Jul 2023

Perceived dignity is an unrecognized source of emotional distress in patients with rheumatic diseases: Results from the validation of the Mexican version of the Patient Dignity Inventory.

PONE-D-23-05892R2

Dear Dr. Pascual-Ramos,

We’re pleased to inform you that your manuscript has been judged scientifically suitable for publication and will be formally accepted for publication once it meets all outstanding technical requirements.

Within one week, you’ll receive an e-mail detailing the required amendments. When these have been addressed, you’ll receive a formal acceptance letter and your manuscript will be scheduled for publication.

An invoice for payment will follow shortly after the formal acceptance. To ensure an efficient process, please log into Editorial Manager at http://www.editorialmanager.com/pone/, click the 'Update My Information' link at the top of the page, and double check that your user information is up-to-date. If you have any billing related questions, please contact our Author Billing department directly at authorbilling@plos.org.

If your institution or institutions have a press office, please notify them about your upcoming paper to help maximize its impact. If they’ll be preparing press materials, please inform our press team as soon as possible -- no later than 48 hours after receiving the formal acceptance. Your manuscript will remain under strict press embargo until 2 pm Eastern Time on the date of publication. For more information, please contact onepress@plos.org.

Kind regards,

Mickael Essouma, M. D.

Academic Editor

PLOS ONE

Additional Editor Comments (optional):

Reviewers' comments:

# Editor report: Acceptance letter

**DOI:** 10.1371/journal.pone.0289315.r006

**Authors:** Mickael Essouma

27 Jul 2023

PONE-D-23-05892R2 

Perceived dignity is an unrecognized source of emotional distress in patients with rheumatic diseases: Results from the validation of the Mexican version of the Patient Dignity Inventory. 

Dear Dr. Pascual-Ramos:

I'm pleased to inform you that your manuscript has been deemed suitable for publication in PLOS ONE. Congratulations! Your manuscript is now with our production department. 

If your institution or institutions have a press office, please let them know about your upcoming paper now to help maximize its impact. If they'll be preparing press materials, please inform our press team within the next 48 hours. Your manuscript will remain under strict press embargo until 2 pm Eastern Time on the date of publication. For more information please contact onepress@plos.org.

If we can help with anything else, please email us at plosone@plos.org. 

Thank you for submitting your work to PLOS ONE and supporting open access. 

Kind regards, 

PLOS ONE Editorial Office Staff

on behalf of

Dr. Mickael Essouma 

Academic Editor

PLOS ONE
